# Logistic regression withstands comparison with machine learning for thyroid nodule malignancy risk stratification under strict temporal validation

**DOI:** 10.1371/journal.pdig.0001828

**Published:** 2026-11-11

**Authors:** Juan Jesús Fernández Alba, Florentino Carral San Laureano, Ana Isabel Jiménez Millán, Carmen González Macías

**Affiliations:** 1 Research Group EM18 "Development and Innovation in Maternal-Fetal Medicine and Gynaecological Oncology", Instituto de Investigación e Innovación Biomédica de Cádiz (INIBICA), Cádiz, Spain; 2 Department of Obstetrics and Gynaecology, Hospital Universitario de Puerto Real, Cádiz, Spain; 3 University of Cadiz, Cádiz, Spain; 4 Department of Endocrinology and Nutrition, Hospital Universitario de Puerto Real, Cádiz, Spain; Xiangtan Central Hospital, CHINA

## Abstract

Ultrasound risk-stratification systems (ACR TI-RADS, EU-TIRADS, ATA 2015) guide fine-needle aspiration decisions for thyroid nodules but do not integrate clinical or biochemical information. Multivariable logistic regression (LR) models incorporating these variables provide individualised malignancy probability estimates, and machine learning (ML) algorithms have been proposed as potential improvements, but rigorous comparative evidence under temporal validation is scarce. We conducted a single-centre retrospective cohort study of 861 thyroidectomies (2010–2023), split chronologically (training: before 2019, n = 432, 94 malignant; test: 2019 onwards, n = 429, 92 malignant). An LR model using the specification of a previously published and externally validated model — with coefficients re-estimated in the training cohort — was compared head-to-head with Random Forest (RF), XGBoost (XGB), Bagged CART, and a multilayer perceptron (MLP) trained on the same predictors. Performance was assessed by discrimination (ROC-AUC and precision-recall AUC), calibration (Brier score, calibration slope and intercept), and clinical utility (decision curve analysis), with operating characteristics reported at the pre-specified 9.55% probability threshold. LR achieved an AUC of 0.838 (95% CI 0.789–0.884), closely matching the published external validation of the original model (0.84). RF (AUC 0.843, p = 0.76) and XGB (AUC 0.844, p = 0.69) were statistically indistinguishable from LR; Bagged CART (0.821, p = 0.40) was non-significantly inferior; the MLP (0.768, p < 0.001) performed significantly worse. LR showed the highest precision-recall AUC (0.672) and good calibration without post-hoc adjustment (slope 1.12, 95% CI 0.91–1.43). LR, RF, XGB and Bagged CART discriminated significantly better than the ATA 2015 classification (AUC 0.750; DeLong p ≤ 0.002), whereas the MLP did not differ significantly from it (p = 0.45). In exploratory subgroup analyses by Bethesda category, nodule size, and age, no evidence of a consistent ML advantage was observed, although several subgroups were underpowered. SHAP importance rankings from RF and XGB showed broadly similar global orderings to the LR coefficients (ρ = 0.71 and 0.77). In this single-centre surgical cohort with a moderate sample size and routinely collected structured predictors, none of the evaluated ML algorithms demonstrated improved performance over a well-specified LR model.

## Author summary

Thyroid nodules are extremely common, yet only a minority are cancerous. Distinguishing the few cancers from the many benign nodules without unnecessary biopsies or surgery is a daily clinical challenge. Current ultrasound-based scores help, but they ignore the patient's age, family history, and blood tests. Our group previously developed and externally validated a simple statistical formula — a logistic regression model — that combines these clinical details with ultrasound features. In this study we asked whether modern machine-learning algorithms could do better when given exactly the same information. We trained four such algorithms on patients operated before 2019 and tested them, together with the regression formula, on patients operated from 2019 onwards — mimicking how a model faces new patients in practice. None of the machine-learning algorithms outperformed the simple formula, and the formula was also better calibrated. Most models discriminated better than the ultrasound-only classification. Our results, obtained in a single-centre surgical cohort of moderate size, suggest that for routine structured clinical data a transparent and interpretable statistical model remains a very strong option — an important consideration under emerging regulations on medical artificial intelligence.

## Introduction

Thyroid nodules are among the most common endocrine findings, detected in up to 65% of adults evaluated by high-resolution ultrasound [1]. The fundamental clinical challenge is accurate risk stratification: identifying the 7–15% of nodules that harbour malignancy while sparing the majority from unnecessary fine-needle aspiration cytology (FNAC) and surgery [1,2]. Current practice relies heavily on ultrasound risk-stratification systems — the 2015 American Thyroid Association (ATA) guidelines, ACR TI-RADS, and EU-TIRADS [2–4] — which classify nodules based on sonographic features. While these systems have improved standardisation, they rely exclusively on ultrasound characteristics and do not integrate the clinical or biochemical information routinely available during patient evaluation, such as age, family history of thyroid cancer, or serum TSH.

Multivariable logistic regression (LR) models that combine clinical, sonographic, and biochemical variables address this gap by providing individualised malignancy probability estimates. Our group previously developed and internally validated such a model [5], which subsequently underwent external validation with decision curve analysis, achieving an AUC of 0.84 and a clinically actionable decision threshold at a 9.55% predicted probability [6].

The proliferation of machine learning (ML) in medical research has generated interest in whether more complex algorithms might improve upon validated LR models for thyroid nodule risk stratification, and several studies have compared ML with LR in this setting, generally finding similar or slightly lower performance for ML [7–9]. However, most comparisons rely on internal cross-validation only, use different predictor sets for different models, or lack temporal validation. The systematic review by Christodoulou and colleagues [10] found no overall advantage of ML over LR across 71 clinical prediction studies; although that review predates current reporting standards and many of the weaknesses it documents reflect the practices of the time, more recent methodological work continues to show that for structured tabular clinical data, well-specified regression models frequently match more complex alternatives [11,12]. Beyond performance, the choice between transparent and black-box models has growing regulatory implications under the EU Artificial Intelligence Act, which classifies clinical decision support tools as high-risk systems requiring transparency and ongoing monitoring [13].

The aim of this study was to determine whether four widely used ML algorithms — Random Forest, XGBoost, Bagged CART, and a multilayer perceptron — improve upon a logistic regression model with a previously validated specification for predicting thyroid nodule malignancy, when all models are trained on identical routinely collected predictors and evaluated under strict temporal validation, with the ATA 2015 classification as a clinical reference.

## Materials and methods

### Ethics statement

This retrospective study was approved by the Biomedical Research Ethics Committee of Cádiz (Spain; protocol code PAI-TIROIDES-2018, April 2018) and conducted in accordance with the Declaration of Helsinki. Owing to the retrospective design, the committee waived the requirement for study-specific informed consent for the use of registry data; all patients had provided written informed consent for the fine-needle aspiration and surgical procedures.

### Study design and participants

We conducted a single-centre retrospective cohort study at Hospital Universitario de Puerto Real (Cádiz, Spain). All consecutive adult patients who underwent thyroidectomy between January 2010 and December 2023 with available histopathological confirmation were eligible. Patients were excluded if model predictors were incomplete. The final complete-case cohort comprised 861 patients. Missing-data analysis confirmed values were distributed at random with respect to the outcome (maximum missingness 6.93% for TSH), supporting complete-case analysis as the primary approach; multiple imputation by chained equations (MICE) was used as a sensitivity analysis. The intended target population of the models evaluated here is patients with thyroid nodules being considered for surgical referral in settings comparable to the derivation centre; applicability to unselected nodule populations managed conservatively is addressed in the Discussion.

### Outcome and predictors

The primary outcome was histopathologically confirmed thyroid malignancy in the surgical specimen (any thyroid cancer), consistent with the outcome analysed in the derivation study [5]; the reference standard was therefore the definitive post-surgical histology of the resected gland. Each patient contributed a single index nodule, defined as the most sonographically suspicious nodule identified at the pre-surgical ultrasound evaluation and recorded as such in the prospective registry; its ultrasound characteristics constitute the model predictors. Not all index nodules underwent fine-needle aspiration (cytology was available for 91.4% of patients). Because the outcome is specimen-level, incidental carcinomas — predominantly papillary microcarcinomas <1 cm detected outside the index nodule — count as events in the primary analysis (16 of the 92 test-cohort events); a sensitivity analysis excluding incidental microcarcinomas, matching the outcome definition applied in the published external validation [6], is reported in Results. The registry antedates the introduction of the NIFTP category; follicular tumours of uncertain malignant potential (n = 4 in the full cohort) were classified as non-malignant.

The predictor set replicated exactly the specification of the published model (Table 1 of [6]): family history of thyroid cancer, male sex, age (with a quadratic term), suppressed TSH (<0.369 µU/mL), elevated TSH (>4.701 µU/mL), autoimmune thyroiditis, solid composition, suspicious cervical adenopathies, hypoechogenicity, microlobed or irregular margins, macrocalcifications, microcalcifications, and taller-than-wide orientation — twelve clinical predictors represented by fourteen model terms (S4 Table). The identical design matrix was supplied to all five models. Bethesda cytology category and ATA 2015 ultrasound risk classification were extracted for subgroup analyses and the clinical baseline comparison; they were not model predictors.

**Table 1:** Baseline characteristics of the training (before 2019) and test (2019 onwards) cohorts. Continuous variables: median [IQR]; categorical: n (%).

Variable	Train (n = 432)	Test (n = 429)	Test statistic	p-value
Age, median [IQR] (years)	50 [41–59]	52 [42–60]	Mann-Whitney U	0.214
Male sex, n (%)	71 (16.4%)	85 (19.8%)	Chi-square	0.198
Family history of thyroid cancer, n (%)	17 (3.9%)	14 (3.3%)	Chi-square	0.597
Serum TSH < 0.369 µU/mL, n (%)	81 (18.8%)	125 (29.1%)	Chi-square	<0.001
Serum TSH > 4.701 µU/mL, n (%)	17 (3.9%)	1 (0.2%)	Chi-square	<0.001
Autoimmune thyroiditis, n (%)	109 (25.2%)	78 (18.2%)	Chi-square	0.012
Nodule diameter, median [IQR] (mm)	31 [21–39]	32 [22–41]	Mann-Whitney U	0.045
≤10 mm, n (%)	12 (2.8%)	15 (3.5%)	Chi-square (4 groups)*	0.002
11–20 mm, n (%)	91 (21.1%)	80 (18.6%)		
21–40 mm, n (%)	253 (58.6%)	212 (49.4%)		
>40 mm, n (%)	76 (17.6%)	122 (28.4%)		
Solid nodule composition, n (%)	315 (72.9%)	327 (76.2%)	Chi-square	0.265
Suspicious cervical lymph nodes, n (%)	34 (7.9%)	29 (6.8%)	Chi-square	0.532
Hypoechoic nodule, n (%)	177 (41.0%)	148 (34.5%)	Chi-square	0.050
Irregular/microlobulated margins, n (%)	65 (15.0%)	44 (10.3%)	Chi-square	0.035
Macrocalcifications, n (%)	43 (10.0%)	42 (9.8%)	Chi-square	0.936
Microcalcifications, n (%)	82 (19.0%)	45 (10.5%)	Chi-square	<0.001
Taller-than-wide orientation, n (%)	47 (10.9%)	27 (6.3%)	Chi-square	0.016
Bethesda cytology category†, n (%)				
Bethesda I (Non-diagnostic)	48 (11.9%)	49 (12.8%)	Chi-square (6 groups)†	0.025
Bethesda II (Benign)	192 (47.5%)	211 (55.1%)		
Bethesda III (AUS/FLUS)	27 (6.7%)	32 (8.4%)		
Bethesda IV (Follicular neoplasm)	85 (21.0%)	47 (12.3%)		
Bethesda V (Suspicious)	32 (7.9%)	30 (7.8%)		
Bethesda VI (Malignant)	20 (5.0%)	14 (3.7%)		
Missing, n (%)	28 (6.5%)	46 (10.7%)		
ATA 2015 ultrasound risk‡, n (%)				
Low suspicion	245 (57.4%)	272 (63.8%)	Chi-square (3 groups)‡	0.017
Intermediate suspicion	61 (14.3%)	69 (16.2%)		
High suspicion	121 (28.3%)	85 (20.0%)		
Thyroid malignancy (outcome), n (%)	94 (21.8%)	92 (21.4%)	Chi-square	0.911

† Computed on patients with cytology available (train: n = 404; test: n = 383). ‡ Computed on patients with ATA data available (train: n = 427; test: n = 426). Standardised mean differences for all predictors are provided in S5 Table.

### Temporal validation design

The cohort was split chronologically: patients evaluated before 2019 formed the training set (n = 432; 94 malignant, 21.8%) and those from 2019 onwards the test set (n = 429; 92 malignant, 21.4%). Table 1 shows the characteristics of both cohorts; standardised mean differences (SMDs) between cohorts are provided in S5 Table. The largest temporal shifts (|SMD| > 0.10) concerned TSH categories (SMD −0.25/ + 0.26), microcalcifications (+0.24), autoimmune thyroiditis (+0.17), taller-than-wide orientation (+0.16), irregular margins (+0.14) and hypoechogenicity (+0.13), reflecting the progressive adoption of EU-TIRADS criteria; outcome prevalence was stable (SMD 0.008).

### Handling of the logistic regression model

The LR model evaluated here uses the predictor specification of the previously published model [5], externally validated in [6]; its coefficients and intercept were re-estimated in the pre-2019 training cohort. This design choice makes the comparison with the ML algorithms symmetric — every model, including LR, was fitted exclusively on the training cohort and evaluated once on the temporal test cohort. Consequently, the present analysis should not be interpreted as an independent validation of the original published model with locked coefficients, but as a temporally validated comparison of modelling algorithms sharing a common, previously validated specification. The re-estimated coefficients are provided in S6 Table and in the public repository. Of note, the training period overlaps with the derivation cohort of the original model [5] and the test period overlaps with the cohort of its external validation [6]; this overlap is acknowledged as a limitation.

### Models and clinical baseline

Five models were trained on the training cohort with identical predictor matrices: (i) LR (maximum likelihood, no penalisation); (ii) Random Forest; (iii) XGBoost; (iv) Bagged CART; and (v) a multilayer perceptron (MLP). Hyperparameters for the ML models were tuned exclusively within the training cohort using 5-fold stratified cross-validation (S2 Table). The four non-linear models were calibrated with isotonic regression implemented via 5-fold cross-validation within the training cohort (CalibratedClassifierCV, scikit-learn), so that the calibration mapping was learned from out-of-fold predictions and no test information was used. The restriction of all models to the same predictor set was a deliberate design decision: it isolates the effect of the learning algorithm while holding the available information constant. Whether an ML pipeline with a broader candidate feature pool could outperform the published specification is a distinct question, addressed in the Discussion. As clinical baseline, the ATA 2015 ultrasound classification was evaluated on the test subset with a valid three-level classification available (n = 426), coding the categories as an ordinal score (very low/low suspicion = 0, intermediate = 1, high = 2) for AUC estimation; this evaluates the discriminatory information of the ATA categories rather than the complete ATA management pathway. ATA categories had been assigned by the treating endocrinologists during routine pre-surgical ultrasound evaluation and recorded in the registry prospectively, before surgery and independently of the histopathological outcome. A sensitivity analysis using the full five-level ATA pattern classification yielded consistent results.

### Performance evaluation

Discrimination was assessed with the area under the ROC curve (AUC; DeLong tests for paired comparisons versus LR and versus the ATA ordinal score [14]) and, given the 21% outcome prevalence, with the area under the precision-recall curve (PR-AUC, average precision) with 2,000-sample bootstrap 95% CIs. Calibration was assessed with the Brier score (paired bootstrap comparisons versus LR), calibration plots, and calibration slope and intercept (calibration-in-the-large, estimated by logistic recalibration with the linear predictor as offset) with bootstrap 95% CIs. Clinical utility was assessed by decision curve analysis across the 5–25% threshold range [15]. Operating characteristics (sensitivity, specificity, PPV, NPV) were computed for all models at the pre-specified 9.55% probability threshold from the external validation of the original model [6] as the primary operating point; Youden-derived thresholds, which are selected post hoc from the test data, are reported as secondary and descriptive only. For ATA 2015, operating characteristics were computed at the standard binary threshold (high suspicion vs. rest).

### Exploratory subgroup analyses

Pre-specified but exploratory subgroup analyses were performed on the test cohort by Bethesda category (I–VI; III–IV pooled as indeterminate; V–VI pooled as suspicious/malignant), nodule size (≤10, 11–20, 21–40, > 40 mm), and age (≤40, 41–55, > 55 years), with AUC and 1,000-bootstrap 95% CIs. Subgroups with fewer than five events or non-events were not analysed. Malignant and benign counts for every subgroup are reported in S3 Table. Given the limited number of events in several strata, these analyses assess the consistency of the main finding and cannot establish equivalence.

### Predictor importance (SHAP)

SHAP values [16] were computed for RF and XGB on the test cohort. Mean absolute SHAP values were ranked and compared descriptively with the ranking of standardised LR coefficient magnitudes using Spearman correlation. This comparison addresses global importance ordering only; it does not test for non-linearities or interactions, and SHAP values and regression coefficients are not directly equivalent quantities.

SHAP values were additionally computed for the LR model itself (LinearExplainer) to enable a like-for-like comparison of global importance profiles across the three approaches.

### Sensitivity analyses

Sensitivity analyses comprised: repeated 10-fold cross-validation (5 repeats); MICE imputation (m = 20) with all five models re-trained and evaluated on each imputed dataset and predictions pooled across imputations; a learning-curve analysis re-fitting every model on stratified random subsamples of 25%, 50%, 75% and 100% of the training cohort (20 replicates per fraction) and evaluating each fit on the fixed temporal test cohort; a resampling sensitivity analysis re-training RF, XGBoost and the MLP after SMOTE oversampling of the training cohort, and with class-weighting (class_weight = ’balanced’ for RF; scale_pos_weight for XGBoost), always evaluating on the intact test cohort [12]; LR with restricted cubic splines for age and pre-specified interactions; and the five-level ATA coding. Full results are in S1 Table and S1 Fig. Analyses were performed in R (4.3) and Python (3.11).

### Reporting and reproducibility

The study is reported per the TRIPOD+AI 2024 statement [17]; the completed checklist is provided as Supporting Information (S1 Checklist). All analysis code, hyperparameter configurations, the re-estimated LR coefficients, and a synthetic dataset enabling full pipeline execution are openly available under an MIT licence at https://github.com/ginejuan/thyroid-ml-vs-lr. The published model is implemented as a free web calculator at https://obgynreference.shinyapps.io/calccdt/. Patients and the public were not involved in the design, conduct, reporting, or dissemination of this study.

## Results

### Study population

Of 1,013 consecutive thyroidectomies, 861 patients were included (Fig 1). Training and test cohorts were balanced for malignancy prevalence (21.8% vs 21.4%, p = 0.911; SMD 0.008), age, sex, and family history (Table 1, S5 Table). Temporal shifts were observed in TSH distribution (suppressed TSH: 18.8% vs 29.1%, p < 0.001), nodule size >40 mm (17.6% vs 28.4%, p = 0.002), microcalcifications (19.0% vs 10.5%, p < 0.001), and ATA high-suspicion category (28.3% vs 20.0%, p = 0.017), reflecting evolving practice over the study period.

**Fig 1:**
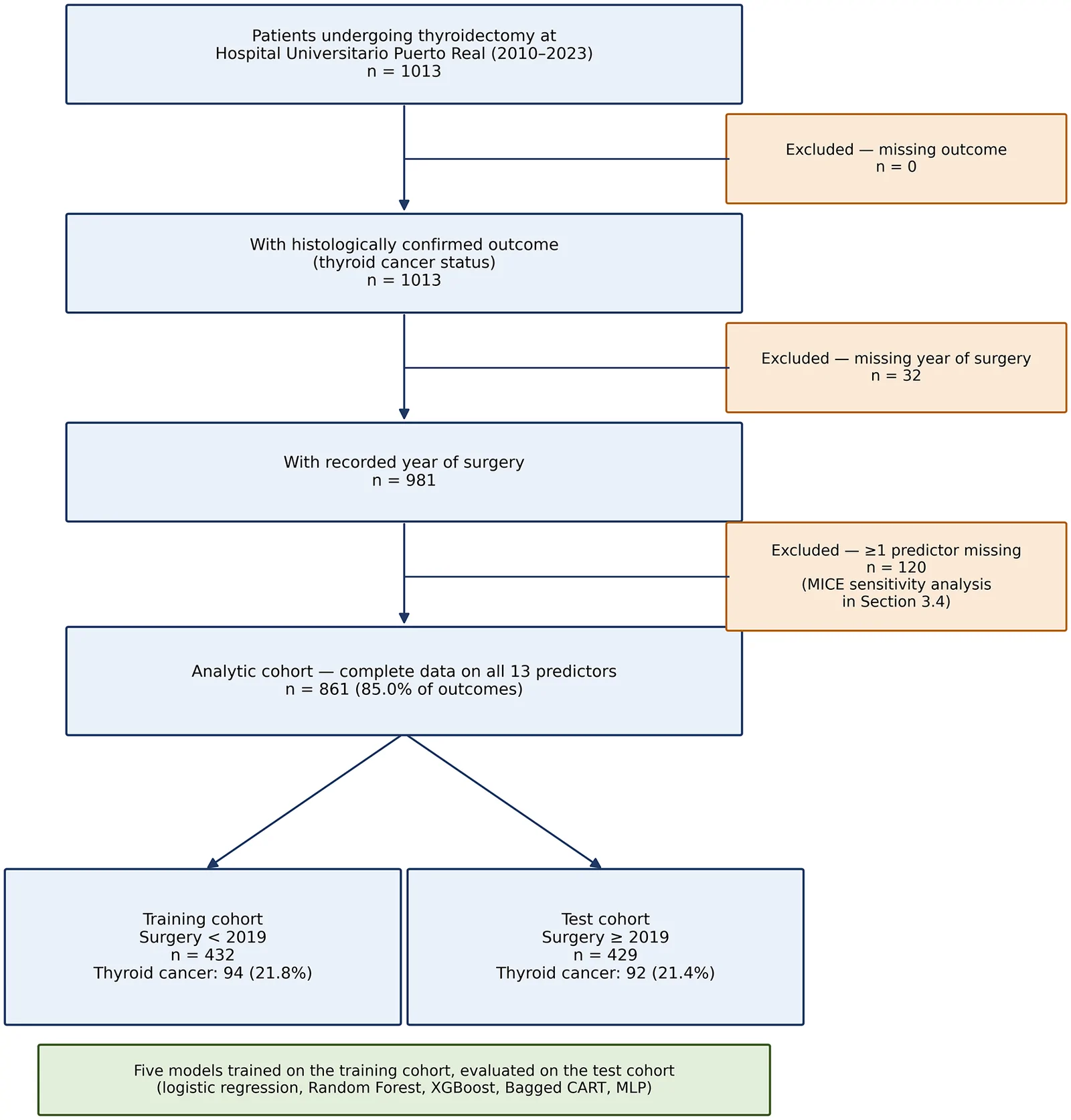
Flow diagram of patient inclusion (1,013 thyroidectomies; 861 included; temporal split 432/429).

### Discrimination and calibration

The re-estimated LR achieved an AUC of 0.838 (95% CI 0.789–0.884) in the temporal test cohort, closely matching the published external validation of the original model (0.84) [6]. No ML algorithm demonstrated improved discrimination (Table 2, Fig 2): RF 0.843 (DeLong p = 0.76), XGB 0.844 (p = 0.69), Bagged CART 0.821 (p = 0.40), MLP 0.768 (p < 0.001, significantly worse). LR additionally showed the highest PR-AUC (0.672, 95% CI 0.574–0.758). LR was the only model requiring no post-hoc calibration and showed the lowest Brier score (0.117) with calibration slope 1.12 (95% CI 0.91–1.43) and intercept 0.42 (0.15–0.67); XGBoost, despite isotonic calibration, showed a slope of 1.42 (1.10–1.93). Decision curves were superimposable across the 5–25% threshold range (Fig 3), indicating no additional net clinical benefit from any ML algorithm; small differences in AUC did not translate into differences in net benefit.

**Table 2:** Model performance on the independent temporal test cohort (n = 429; 92 malignant, 21.4%).

Model	AUC (95% CI)	DeLong p vs LR	PR-AUC (95% CI)	Brier	Cal. slope (95% CI)	Cal. intercept (95% CI)
Logistic Regression	0.838 (0.789–0.884)	—	0.672 (0.574–0.758)	0.117	1.12 (0.91–1.43)	0.42 (0.15–0.67)
Random Forest	0.843 (0.795–0.886)	0.76	0.637 (0.535–0.730)	0.119	1.06 (0.86–1.35)	0.36 (0.08–0.62)
XGBoost	0.844 (0.802–0.886)	0.69	0.625 (0.524–0.727)	0.126	1.42 (1.10–1.93)	0.34 (0.08–0.58)
Bagged CART	0.821 (0.774–0.868)	0.40	0.594 (0.485–0.697)	0.129	1.32 (1.02–1.75)	0.39 (0.13–0.64)
MLP	0.768 (0.707–0.827)	<0.001	0.591 (0.490–0.680)	0.131	1.12 (0.83–1.49)	0.23 (−0.03–0.47)

AUC: area under the ROC curve; PR-AUC: area under the precision-recall curve (average precision; prevalence 21.4%). Calibration slope and intercept estimated by logistic recalibration on the test cohort; 95% CIs from 2,000-sample bootstrap. Paired bootstrap Brier comparisons vs LR: RF p = 0.60; XGB p = 0.03; Bagged CART p < 0.001. The four non-linear models received isotonic calibration (5-fold CV within training); LR received no post-hoc calibration.

**Fig 2:**
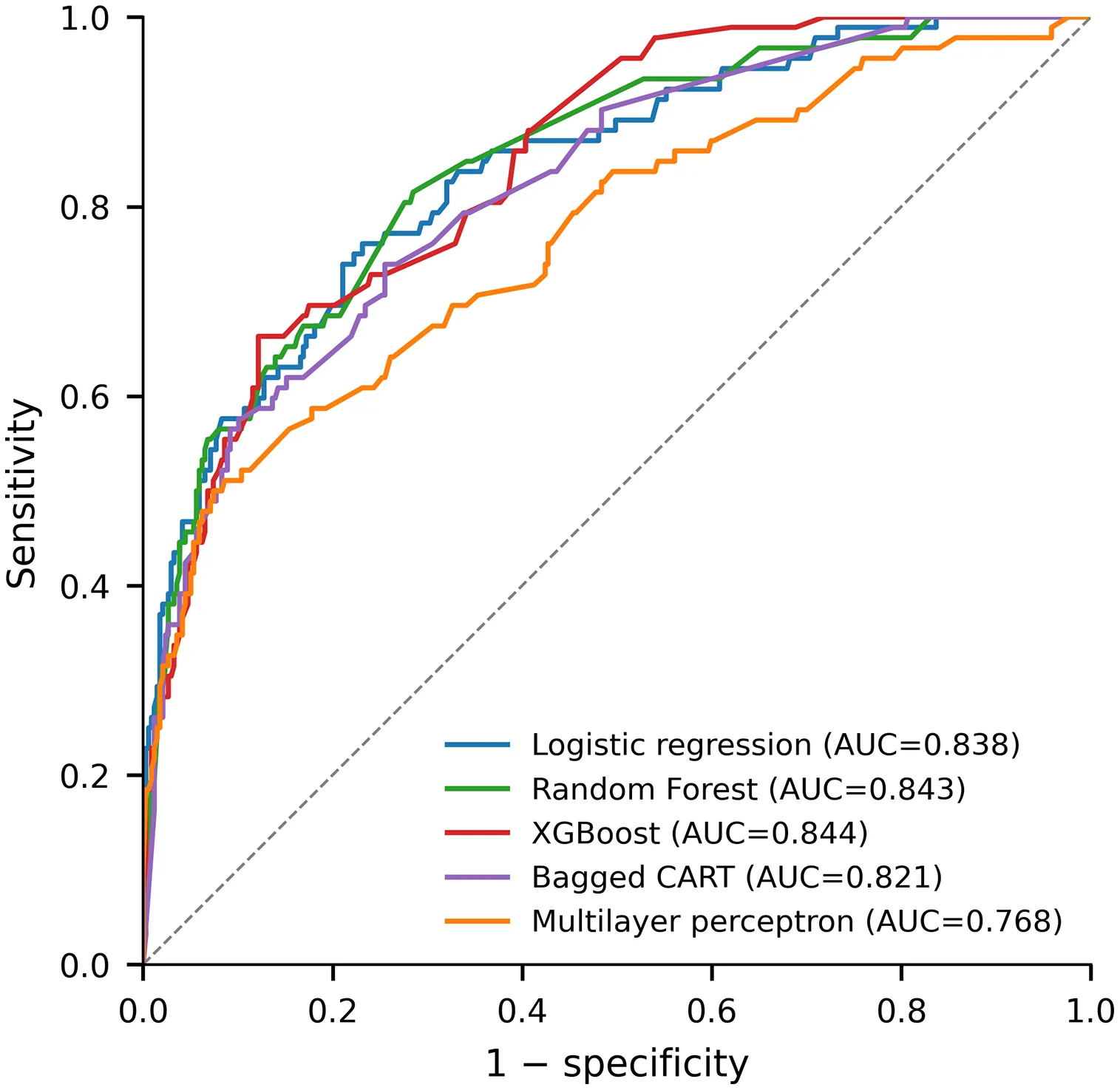
ROC curves of the five models on the temporal test cohort (n = 429).

**Fig 3:**
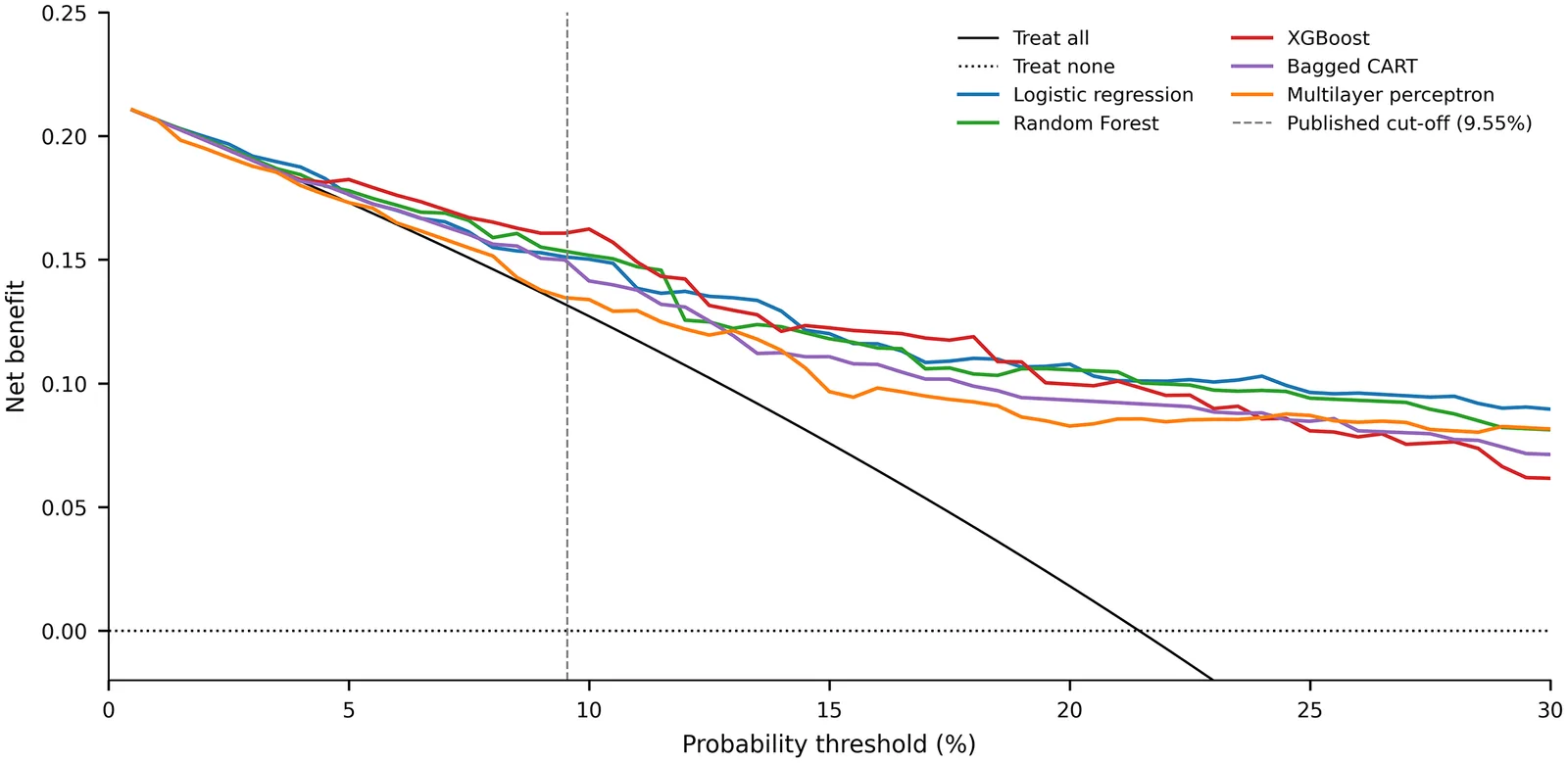
Decision curves across the 5–25% threshold range, temporal test cohort.

### Operating characteristics at the pre-specified threshold

At the pre-specified 9.55% threshold [6] — the primary operating point — LR achieved sensitivity 0.859 (95% CI 0.780–0.929), specificity 0.599 (0.547–0.651), PPV 0.369 and NPV 0.940 (0.905–0.969) (Table 3). RF showed comparable sensitivity (0.848, 95% CI 0.771–0.916) and NPV (0.940, 0.909–0.967), with higher specificity (0.656 vs 0.599) and PPV (0.402 vs 0.369) than LR; XGBoost operated as a nearly rule-out classifier at this threshold (sensitivity 0.989, specificity 0.377). Youden-derived thresholds, being test-set-optimised, are reported as descriptive secondary results in the table footnote.

**Table 3:** Operating characteristics at the pre-specified 9.55% probability threshold (primary operating point) and for the ATA 2015 binary classification, temporal test cohort.

Classifier	Threshold	Sensitivity (95% CI)	Specificity (95% CI)	PPV	NPV (95% CI)
Logistic Regression	9.55% (pre-specified)	0.859 (0.780–0.929)	0.599 (0.547–0.651)	0.369	0.940 (0.905–0.969)
Random Forest	9.55%	0.848 (0.771–0.916)	0.656 (0.606–0.706)	0.402	0.940 (0.909–0.967)
XGBoost	9.55%	0.989 (0.964–1.000)	0.377 (0.326–0.429)	0.302	0.992 (0.975–1.000)
Bagged CART	9.55%	0.880 (0.806–0.940)	0.531 (0.478–0.584)	0.339	0.942 (0.906–0.972)
MLP	9.55%	0.891 (0.821–0.950)	0.315 (0.268–0.365)	0.262	0.914 (0.857–0.963)
ATA 2015 (high suspicion)	binary	0.522	0.889	0.565	0.871

95% CIs from 2,000-sample bootstrap. Secondary, test-set-derived Youden thresholds (descriptive only): LR 13.4% (sens 0.761/spec 0.769); RF 11.5% (0.815/0.715); XGB 17.8% (0.663/0.878); Bagged CART 12.4% (0.739/0.745); MLP 26.4% (0.500/0.926).

### Comparison with ATA 2015 risk classification

On the test subset with a valid three-level ATA classification (n = 426; 92 malignant), the ATA 2015 ordinal score achieved an AUC of 0.750 (95% CI 0.693–0.806). LR, RF, XGBoost and Bagged CART discriminated significantly better than ATA 2015 (paired DeLong p < 0.001, p < 0.001, p < 0.001 and p = 0.002, respectively), whereas the MLP (AUC 0.767) did not differ significantly from it (p = 0.45) (Table 4). A sensitivity analysis using the five-level ATA pattern classification (n = 429, AUC 0.766) yielded the same conclusions (p ≤ 0.021 for the four main models; p = 0.95 for the MLP). These results indicate that adding clinical and biochemical predictors to sonographic information improves discrimination over sonographic-only classification, while ATA/TI-RADS systems retain their distinct roles in standardised reporting and management guidance; the prediction models are proposed as complements, not replacements. Note: the ATA AUC of 0.779 reported in the originally submitted version could not be reproduced during this revision and has been corrected to the verified value of 0.750 (three-level ordinal coding).

**Table 4:** Discrimination of prediction models vs. ATA 2015 ultrasound risk classification (three-level ordinal coding), test subset with valid ATA classification (n = 426; 92 malignant).

Classifier	AUC (95% CI)	DeLong p vs ATA
ATA 2015 (three-level ordinal)	0.750 (0.693–0.806)	—
Logistic Regression	0.837	<0.001
Random Forest	0.842	<0.001
XGBoost	0.843	<0.001
Bagged CART	0.821	0.002
MLP	0.767	0.45

Paired DeLong tests on the common subset. Sensitivity analysis with the five-level ATA pattern classification (n = 429): ATA AUC 0.766 (0.713–0.818); model comparisons unchanged (LR p < 0.001; RF p < 0.001; XGB p < 0.001; Bagged CART p = 0.021; MLP p = 0.95).

### Exploratory subgroup analyses

No evidence of a consistent ML advantage over LR was observed in any subgroup (S3 Table, which reports malignant and benign counts for every stratum). In the clinically relevant Bethesda III–IV indeterminate subgroup (n = 79; 34 malignant), LR achieved AUC 0.720 (0.599–0.827) vs RF 0.707 and XGB 0.691. In the Bethesda V–VI subgroup (n = 44; 36 malignant, 8 benign) all models showed AUC ≈ 0.61–0.65 with very wide CIs; this stratum was underpowered and its estimates are unstable, as is the ≤ 10 mm size stratum (n = 15; 10 malignant), whose apparently excellent AUCs (0.88–0.98) should not be over-interpreted. Across the remaining size subgroups LR achieved AUC 0.837 (11–20 mm), 0.762 (21–40 mm) and 0.796 (>40 mm), and performance was consistent across age subgroups (LR AUC 0.830–0.856). Because absence of a significant difference does not establish equivalence, these exploratory findings should be interpreted as showing no evidence of advantage rather than proof of equality.

### Predictor importance

SHAP importance rankings from RF and XGB showed broadly similar global orderings to the standardised LR coefficients (Spearman ρ = 0.71, p = 0.004 for RF; ρ = 0.77, p = 0.001 for XGB; Fig 4, Table 5). SHAP values computed on the LR model itself correlated strongly with those of XGBoost (ρ = 0.88, p < 0.001) and RF (ρ = 0.69, p = 0.006), and with the standardised-coefficient ranking (ρ = 0.85, p < 0.001), confirming the agreement with a common importance metric. The six top-ranked predictors were the same across the three approaches: microcalcifications, hypoechogenicity, age, suppressed TSH, irregular margins, and suspicious lymph nodes (with solid composition and autoimmune thyroiditis also ranking consistently). This agreement in global ranking is descriptive; it does not establish identical predictor hierarchies nor exclude non-linear effects.

**Table 5:** Predictor importance ranking: standardised LR coefficients vs. SHAP mean absolute values (RF, XGBoost).

Rank	Logistic Regression	Random Forest (SHAP)	XGBoost (SHAP)
1	Microcalcifications	Microcalcifications	Microcalcifications
2	Hypoechogenicity	Hypoechogenicity	Hypoechogenicity
3	Age	Age	Age
4	Low TSH (<0.369 µU/mL)	Low TSH	Irregular margins
5	Irregular margins	Irregular margins	Low TSH
6	Suspicious lymph nodes	Suspicious lymph nodes	Suspicious lymph nodes

SHAP: SHapley Additive exPlanations. Spearman rank correlations: LR vs RF ρ = 0.71; LR vs XGB ρ = 0.77. Rankings are broadly similar across approaches; the comparison is descriptive.

**Fig 4:**
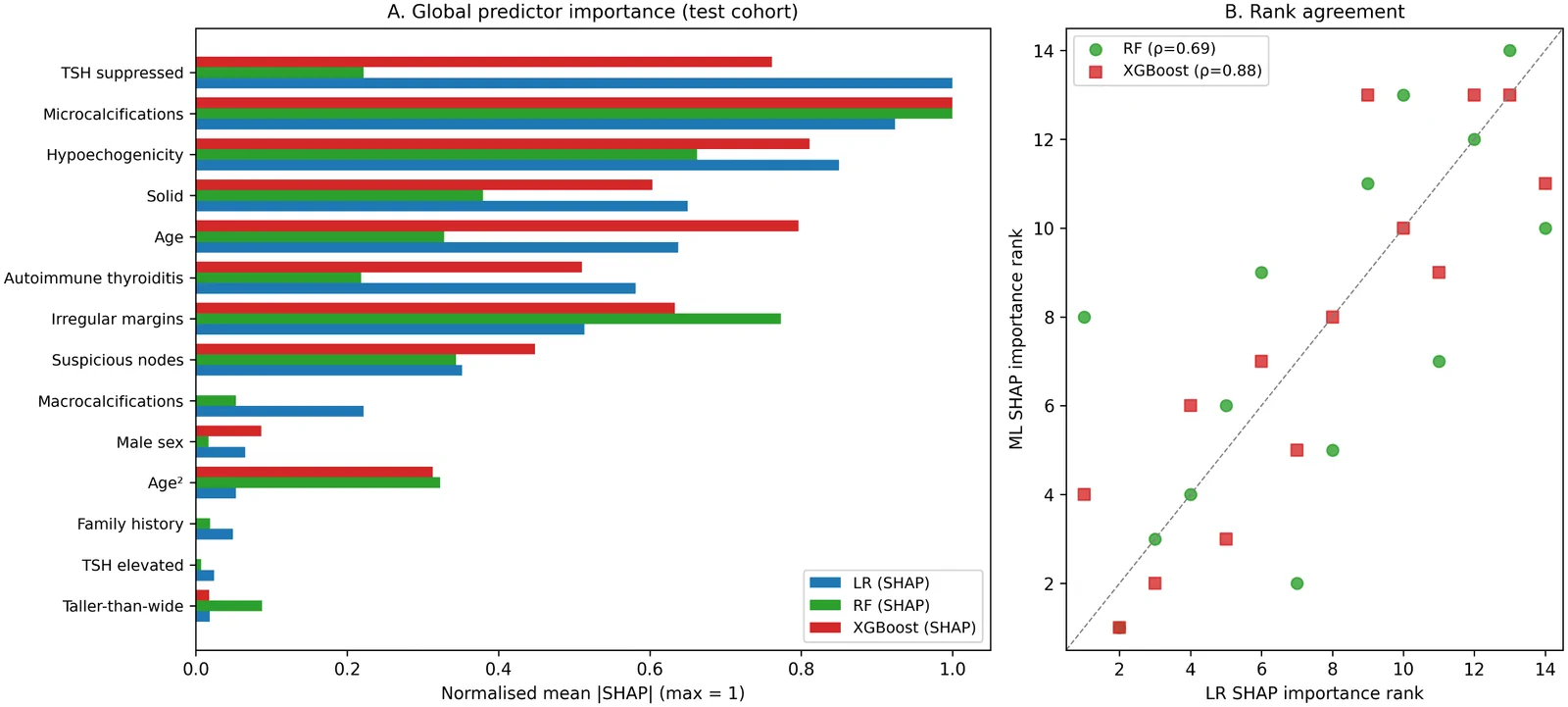
Predictor importance. **(A)** Normalised mean absolute SHAP values on the temporal test cohort for LR (LinearExplainer), Random Forest and XGBoost. **(B)** Rank agreement between LR and ML importance orderings (Spearman ρ: RF 0.69; XGBoost 0.88).

### Sensitivity analyses

Repeated cross-validation confirmed that LR achieved the lowest and most stable Brier score across folds. Under MICE imputation (m = 20; n = 499 training, n = 482 test), pooled test AUCs were 0.831 (LR), 0.839 (RF), 0.845 (XGB), 0.810 (Bagged CART) and 0.816 (MLP), reproducing the complete-case pattern of no ML advantage; the MLP again showed the largest between-imputation instability (individual-imputation AUC range 0.70–0.84). Learning curves (S1 Fig) showed that RF and XGBoost had largely plateaued at the full training size (mean AUC gain from 75% to 100% of the training cohort: + 0.005 and +0.008, respectively), whereas the MLP was still clearly data-limited (AUC 0.663 → 0.785 from 25% to 100%), indicating that additional data of the same kind would be unlikely to reverse the tree-ensemble results but might benefit the neural network. In the resampling sensitivity analysis, SMOTE increased the RF AUC slightly (0.843 → 0.858) at the cost of clear calibration deterioration (slope 1.06 → 0.79; mean predicted risk 0.239 vs observed prevalence 0.214), degraded XGBoost on all metrics (AUC 0.844 → 0.834; slope 1.42 → 0.61), and left the MLP essentially unchanged in discrimination while worsening its Brier score; class-weighting produced no improvements. These findings are consistent with methodological work showing that imbalance corrections tend to harm risk-prediction calibration [12]. LR with restricted cubic splines and interactions did not improve on the main specification (age linearity test p = 0.191). The five-level ATA coding did not alter the ATA comparison. Full results in S1 Table. In the outcome-definition sensitivity analysis — excluding incidental microcarcinomas, as in the published external validation [6] — discrimination improved for every model and LR achieved the highest AUC (LR 0.887; XGB 0.875; RF 0.869; Bagged CART 0.859; MLP 0.821; 76 events), consistent with incidental microcarcinomas being essentially unpredictable from index-nodule characteristics; the same pattern was obtained using the registry's differentiated-carcinoma outcome excluding microcarcinomas (LR 0.883). The primary conclusion was unchanged under every sensitivity analysis.

## Discussion

In this single-centre surgical cohort with a moderate sample size and twelve routinely collected structured predictors, none of the four evaluated machine-learning algorithms demonstrated improved performance over a logistic regression model sharing the same, previously validated specification, under strict temporal validation. The re-estimated LR reproduced almost exactly the discrimination reported in the external validation of the original model (AUC 0.838 vs 0.84) across a period in which clinical practice shifted measurably — with standardised mean differences up to 0.26 for TSH categories — and it achieved this with the best calibration profile of all models and without post-hoc adjustment. Notably, the documented distribution shift did not disproportionately degrade the ML models’ discrimination relative to LR, although it may have contributed to the MLP's poor performance. For clinicians and institutions considering ML-based risk stratification tools for thyroid nodules, these findings indicate that in data contexts like ours — clinical cohorts of hundreds of patients with tabular structured predictors, which remain the most common scenario in prediction research — added algorithmic complexity did not translate into better risk stratification.

LR, RF, XGBoost and Bagged CART discriminated significantly better than the ATA 2015 ultrasound classification, whereas the MLP did not. This should not be read as criticism of ATA or TI-RADS systems, whose primary purposes include standardised reporting and management guidance and whose recommendations integrate nodule size and other clinical elements not captured by an ordinal AUC comparison. Rather, the results support formal prediction models as complements that add clinical and biochemical information to sonographic risk stratification.

The exploratory subgroup analyses found no evidence of an ML advantage in any stratum, including the clinically relevant Bethesda III–IV indeterminate group, though several strata had few events and wide confidence intervals; these analyses cannot establish equivalence. The learning-curve analysis adds an important piece of context to the sample-size limitation: the tree ensembles had largely plateaued at the available training size, suggesting that the absence of an RF/XGBoost advantage is not merely an artefact of limited data, whereas the MLP was still clearly data-limited, which likely explains its poor performance. The resampling sensitivity analysis likewise showed that class-imbalance corrections did not provide a genuine improvement: the only AUC gain (RF with SMOTE) came at the cost of a marked calibration deterioration, in line with previous methodological warnings [12]. The SHAP analysis offers a plausible explanation for the overall result: the ML models relied on broadly the same global predictor ordering as the LR coefficients — an agreement confirmed when SHAP was computed on the LR model itself (ρ = 0.69–0.88) — suggesting that with this information set there was little exploitable non-linear signal beyond the specified main effects, a finding consistent with the pre-specified spline and interaction analyses, which likewise showed no improvement.

Our findings align with and extend the literature on tabular clinical data. The systematic review by Christodoulou et al. found no aggregate benefit of ML over LR [10]; while that evidence base predates TRIPOD+AI and reflects earlier methodological standards, more recent work continues to show that for low-dimensional structured data, gradient-boosted trees and neural networks offer no systematic advantage over well-specified regression, and that resampling corrections for class imbalance can even harm calibration [11,12]. ML approaches are most likely to add value with high-dimensional inputs (radiomics, genomics, imaging), very large samples, or strongly non-linear outcome mechanisms [18] — none of which characterise routine thyroid nodule assessment with structured predictors. Future gains are therefore more likely from enriching the predictor set (molecular markers, standardised TI-RADS scoring, elastography, radiomics) than from exchanging the algorithm [19].

The equivalence observed here has practical and regulatory implications. Under the EU AI Act, high-risk clinical AI systems must demonstrate transparency, explainability and ongoing monitoring [13]; a transparent regression model with equivalent performance satisfies these requirements with far less operational burden than black-box alternatives. The published model is deployable as a point-of-care calculator in populations comparable to the derivation setting, with periodic performance audits (e.g., re-evaluation triggered by calibration slope <0.80 or AUC decline >0.05). However, deployment claims must respect the boundary conditions of our design, discussed next.

This study has limitations. First, the cohort includes only patients who underwent thyroidectomy. This verification bias means prevalence (21%), case mix and sonographic spectrum differ from unselected nodule populations; although discrimination may transfer, the predicted probabilities, the decision-curve results and the 9.55% threshold cannot be assumed valid in patients managed with FNAC or surveillance, and validation in a representative non-surgical cohort is required before use in that setting. Second, the LR coefficients were re-estimated in the training cohort; the analysis is therefore a symmetric comparison of algorithms, not an independent validation of the original locked model, and the training and test periods overlap with the cohorts of the original derivation [5] and external validation [6] studies respectively. Third, the training sample (n = 432, 94 events; ~ 7 events per model term) is below recommended minima for prediction modelling [20] and is limited for data-intensive algorithms; the absence of an observed ML advantage may partly reflect restricted training information, and our results should not be extrapolated to substantially larger datasets. Fourth, restricting all models to the published predictor specification was a deliberate design choice that isolates the algorithm effect but may cap the achievable performance of ML pipelines with broader feature pools; whether such pipelines could outperform the published specification is a separate question for future research. Fifth, several subgroup analyses were underpowered. Sixth, the study does not address image-based deep learning, a fundamentally different paradigm. Finally, demographic attributes beyond age and sex were unavailable, precluding formal fairness analyses.

## Conclusions

In a single-centre surgical cohort with a moderate sample size, four machine-learning algorithms trained on the same twelve routinely collected predictors did not demonstrate improved discrimination, calibration or clinical utility over a logistic regression model with a previously validated specification under strict temporal validation, and all models except the MLP discriminated significantly better than the ATA 2015 ultrasound classification. Within these boundary conditions, a transparent regression model remains a strong default for thyroid nodule malignancy risk stratification with structured clinical data, pending validation in non-surgical populations and exploration of enriched predictor sets.

## Supporting information

S1 Table Sensitivity analyses (repeated CV; MICE with all five models; SMOTE/class-weighting; splines; five-level ATA coding). (DOCX)

S2 Table Hyperparameters. (DOCX)

S3 Table Exploratory subgroup analyses with events per stratum. (DOCX)

S4 Table Predictor definitions and coding. (DOCX)

S5 Table Standardised mean differences train vs test. (DOCX)

S6 Table Re-estimated LR coefficients. (DOCX)

S1 Checklist TRIPOD+AI. (DOCX)

S1 Fig Learning curves (AUC vs training-set size, 20 stratified subsamples per fraction). (TIF)
