# Supplementary material for: Logistic regression withstands comparison with machine learning for thyroid nodule malignancy risk stratification under strict temporal validation
Source: PLOS Digit Health. 2026 Nov 11;5(11):e0001828. doi: 10.1371/journal.pdig.0001828 (PMC13754167; doi:10.1371/journal.pdig.0001828)
Supplement: Supplementary file 1 [file pdig.0001828.s001.docx]

**S1 Table. Sensitivity analyses.**

*All sensitivity analyses corroborated the primary finding: none of the evaluated ML algorithms demonstrated improved performance over the re-estimated logistic regression (LR).*

**A. Multiple imputation (MICE, m=20; n=499 training / n=482 test): predictions pooled across imputations.**

| **Model** | **AUC pooled (MICE m=20)** | **Brier pooled** | **AUC range across imputations** |
| --- | --- | --- | --- |
| Logistic Regression | 0.831 | 0.114 | 0.826–0.837 |
| Random Forest | 0.839 | 0.114 | 0.833–0.846 |
| XGBoost | 0.845 | 0.118 | 0.828–0.854 |
| Bagged CART | 0.810 | 0.124 | 0.801–0.833 |
| MLP | 0.816 | 0.117 | 0.697–0.839 |

*The MLP showed the largest between-imputation instability.*

**B. Learning curves: test-cohort AUC (mean ± SD over 20 stratified subsamples) by training-set fraction (see also S1 Fig).**

| **Model** | **25% (n=108)** | **50% (n=216)** | **75% (n=324)** | **100% (n=432)** | **Gain 75%→100%** |
| --- | --- | --- | --- | --- | --- |
| Logistic Regression | 0.780 ± 0.047 | 0.818 ± 0.017 | 0.829 ± 0.013 | 0.838 | +0.009 |
| Random Forest | 0.811 ± 0.019 | 0.833 ± 0.012 | 0.842 ± 0.009 | 0.847 ± 0.002 | +0.005 |
| XGBoost | 0.741 ± 0.052 | 0.812 ± 0.017 | 0.840 ± 0.010 | 0.848 ± 0.002 | +0.008 |
| Bagged CART | 0.787 ± 0.023 | 0.805 ± 0.020 | 0.811 ± 0.020 | 0.802 ± 0.003 | −0.009 |
| MLP | 0.663 ± 0.133 | 0.720 ± 0.115 | 0.775 ± 0.046 | 0.785 ± 0.029 | +0.010 |

*RF and XGBoost were largely plateaued at the full training size; the MLP remained data-limited.*

**C. Resampling sensitivity: SMOTE and class-weighting on the training cohort, evaluated on the intact test cohort.**

| **Variant** | **AUC** | **PR-AUC** | **Brier** | **Calibration slope** |
| --- | --- | --- | --- | --- |
| RF (main analysis) | 0.843 | 0.637 | 0.119 | 1.06 |
| RF + SMOTE | 0.858 | 0.655 | 0.116 | 0.79 |
| RF class_weight='balanced' | 0.838 | 0.630 | 0.123 | 1.08 |
| XGB (main analysis) | 0.844 | 0.625 | 0.126 | 1.42 |
| XGB + SMOTE | 0.834 | 0.604 | 0.129 | 0.61 |
| XGB scale_pos_weight | 0.802 | 0.574 | 0.132 | 1.26 |
| MLP (main analysis) | 0.787 | 0.603 | 0.124 | 1.08 |
| MLP + SMOTE | 0.792 | 0.606 | 0.143 | 0.70 |

*The only AUC gain (RF+SMOTE) came at the cost of clear calibration deterioration (slope 1.06→0.79; mean predicted risk 0.239 vs observed prevalence 0.214).*

**D. Outcome-definition sensitivity (test cohort AUC).**

| **Model** | **Primary outcome (any cancer; 92 events)** | **Excluding incidental microcarcinomas (76 events)** | **Registry DTC excluding microcarcinomas (75 events)** |
| --- | --- | --- | --- |
| Logistic Regression | 0.838 | 0.887 | 0.883 |
| Random Forest | 0.843 | 0.869 | 0.863 |
| XGBoost | 0.844 | 0.875 | 0.869 |
| Bagged CART | 0.821 | 0.859 | 0.854 |
| MLP | 0.768 | 0.821 | 0.818 |

*Excluding incidental microcarcinomas — the outcome definition of the published external validation — improved discrimination for every model, with LR highest.*

**E. Additional specification and robustness checks.**

| **Analysis** | **Result** |
| --- | --- |
| LR with restricted cubic splines for age | No improvement over the age+age² specification (LRT p=0.191) |
| Pre-specified two-way interactions | No significant improvement |
| Five-level ATA pattern coding (n=429) | ATA AUC 0.766 (0.713–0.818); model-vs-ATA conclusions unchanged (four models p≤0.021; MLP p=0.95) |
| Repeated 10-fold CV (5 repeats) | LR lowest and most stable Brier score across folds |
