# Supplementary material for: Logistic regression withstands comparison with machine learning for thyroid nodule malignancy risk stratification under strict temporal validation
Source: PLOS Digit Health. 2026 Nov 11;5(11):e0001828. doi: 10.1371/journal.pdig.0001828 (PMC13754167; doi:10.1371/journal.pdig.0001828)
Supplement: Supplementary file 2 [file pdig.0001828.s002.docx]

**S2 Table. Hyperparameter search spaces and selected configurations.**

*Tuning by 5-fold stratified cross-validation within the training cohort only (optimisation metric: ROC-AUC). The four non-linear models were subsequently calibrated with isotonic regression learned from out-of-fold training predictions (CalibratedClassifierCV, cv=5).*

| **Model** | **Search space (5-fold CV on training set, AUC criterion)** | **Selected configuration** |
| --- | --- | --- |
| **Logistic regression** | No tuning. Penalty = none, solver = lbfgs (max_iter = 2,000). | All 13 published predictors including age² (no penalisation). |
| **Random Forest** | n_estimators = {500}; max_features ∈ {3, 4, 5, 6, sqrt}; min_samples_leaf ∈ {1, 3, 5, 10}; max_depth = None. | n_estimators = 500; max_features = sqrt; min_samples_leaf = 5; max_depth = None. Internal isotonic calibration (5-fold). |
| **XGBoost** | n_estimators ∈ {300, 600}; max_depth ∈ {3, 4, 6}; learning_rate ∈ {0.03, 0.05, 0.10}; subsample = 0.8; colsample_bytree = 0.8; reg_lambda = 1.0; min_child_weight ∈ {1, 5}. | n_estimators = 300; max_depth = 3; learning_rate = 0.05; min_child_weight = 5; tree_method = hist. Internal isotonic calibration (5-fold). |
| **Bagged CART** | n_estimators ∈ {200, 500}; max_depth ∈ {None, 5, 10}; min_samples_leaf ∈ {1, 5, 10}; bootstrap = True. | n_estimators = 500; max_depth = 5; min_samples_leaf = 5. Internal isotonic calibration (5-fold). |
| **MLP** | hidden_layer_sizes ∈ {(8,), (16,), (8, 4), (16, 8)}; alpha (L2) ∈ {1e-3, 1e-2, 1e-1}; learning_rate_init ∈ {1e-3, 1e-2}; activation = ReLU; early stopping (15 epochs without validation improvement); StandardScaler pipeline. | hidden_layer_sizes = (8,); alpha = 1e-2; learning_rate_init = 1e-3. Internal isotonic calibration (5-fold). |
