# Supplementary material for: Logistic regression withstands comparison with machine learning for thyroid nodule malignancy risk stratification under strict temporal validation
Source: PLOS Digit Health. 2026 Nov 11;5(11):e0001828. doi: 10.1371/journal.pdig.0001828 (PMC13754167; doi:10.1371/journal.pdig.0001828)
Supplement: Supplementary file 3 [file pdig.0001828.s003.docx]

**S3 Table. Exploratory subgroup analyses (temporal test cohort).**

*AUC (95% bootstrap CI) by Bethesda category, nodule size and age, with malignant counts per stratum (benign = n − malignant). '—': not analysed (fewer than five events or non-events). Estimates in small strata (Bethesda III, V, V–VI pooled; ≤10 mm) are unstable and exploratory; absence of a significant difference does not establish equivalence.*

| **Subgroup** | **n** | **Malignant n (%)** | **AUC LR** | **AUC RF** | **AUC XGB** | **AUC CART** | **AUC MLP** |
| --- | --- | --- | --- | --- | --- | --- | --- |
| Bethesda I | 49 | 2 (4%) | — | — | — | — | — |
| Bethesda II | 211 | 18 (9%) | 0.683 [0.558–0.803] | 0.782 [0.704–0.874] | 0.760 [0.655–0.862] | 0.723 [0.598–0.820] | 0.649 [0.518–0.767] |
| Bethesda III | 32 | 14 (44%) | 0.639 [0.427–0.836] | 0.567 [0.352–0.785] | 0.571 [0.352–0.773] | 0.591 [0.357–0.804] | 0.631 [0.409–0.827] |
| Bethesda IV | 47 | 20 (43%) | 0.780 [0.642–0.906] | 0.787 [0.654–0.915] | 0.752 [0.602–0.883] | 0.791 [0.649–0.908] | 0.665 [0.494–0.822] |
| Bethesda V | 30 | 24 (80%) | 0.681 [0.428–0.917] | 0.667 [0.412–0.864] | 0.708 [0.477–0.896] | 0.604 [0.340–0.847] | 0.715 [0.486–0.888] |
| Bethesda VI | 14 | 12 (86%) | — | — | — | — | — |
| Bethesda III-IV (Indeterminado) | 79 | 34 (43%) | 0.720 [0.599–0.827] | 0.707 [0.590–0.816] | 0.691 [0.559–0.798] | 0.718 [0.593–0.820] | 0.656 [0.515–0.775] |
| Bethesda V-VI (Sospechoso/Maligno) | 44 | 36 (82%) | 0.646 [0.421–0.844] | 0.646 [0.448–0.840] | 0.653 [0.452–0.830] | 0.608 [0.389–0.840] | 0.632 [0.438–0.815] |
| ≤10 mm | 15 | 10 (67%) | 0.980 [0.889–1.000] | 0.980 [0.852–1.000] | 0.880 [0.643–1.000] | 0.920 [0.727–1.000] | 0.960 [0.778–1.000] |
| 11–20 mm | 80 | 32 (40%) | 0.837 [0.747–0.922] | 0.829 [0.744–0.921] | 0.820 [0.729–0.910] | 0.829 [0.740–0.917] | 0.758 [0.648–0.861] |
| 21–40 mm | 212 | 33 (16%) | 0.762 [0.679–0.847] | 0.767 [0.681–0.855] | 0.797 [0.723–0.870] | 0.766 [0.681–0.848] | 0.673 [0.565–0.783] |
| >40 mm | 122 | 17 (14%) | 0.796 [0.654–0.917] | 0.836 [0.731–0.922] | 0.826 [0.728–0.907] | 0.756 [0.621–0.857] | 0.739 [0.597–0.871] |
| ≤40 años | 93 | 22 (24%) | 0.848 [0.731–0.944] | 0.845 [0.740–0.939] | 0.824 [0.709–0.918] | 0.835 [0.731–0.932] | 0.827 [0.702–0.921] |
| 41–55 años | 165 | 38 (23%) | 0.856 [0.781–0.919] | 0.845 [0.761–0.915] | 0.857 [0.791–0.913] | 0.826 [0.747–0.897] | 0.784 [0.695–0.873] |
| >55 años | 171 | 32 (19%) | 0.830 [0.756–0.897] | 0.837 [0.768–0.902] | 0.830 [0.766–0.896] | 0.796 [0.717–0.876] | 0.718 [0.606–0.815] |
