# Supplementary material for: Logistic regression withstands comparison with machine learning for thyroid nodule malignancy risk stratification under strict temporal validation
Source: PLOS Digit Health. 2026 Nov 11;5(11):e0001828. doi: 10.1371/journal.pdig.0001828 (PMC13754167; doi:10.1371/journal.pdig.0001828)
Supplement: Supplementary file 4 [file pdig.0001828.s004.docx]

**S4 Table. Predictor definitions, coding and transformations.**

*Twelve clinical predictors represented by fourteen model terms, replicating exactly Table 1 of the published external validation (Fernández Alba et al., Diagnostics 2025;15:686). The identical design matrix was supplied to all five models.*

| **#** | **Predictor** | **Type** | **Coding / transformation** | **Model term(s)** |
| --- | --- | --- | --- | --- |
| 1 | Family history of thyroid cancer | Binary | 1 = first-degree family history | fhx_tc |
| 2 | Sex | Binary | 1 = male | male |
| 3 | Age | Continuous | Years; quadratic term included | age, age² |
| 4 | Serum TSH | Categorical (3) | Suppressed (<0.369 µU/mL) / normal (ref) / elevated (>4.701 µU/mL) | tsh_low, tsh_high |
| 5 | Autoimmune thyroiditis | Binary | 1 = present | autoimmune |
| 6 | Solid composition | Binary | 1 = solid nodule | solid |
| 7 | Suspicious cervical adenopathies | Binary | 1 = present | susp_nodes |
| 8 | Hypoechogenicity | Binary | 1 = hypoechoic | hypoechoic |
| 9 | Microlobed or irregular margins | Binary | 1 = present | irregular |
| 10 | Macrocalcifications | Binary | 1 = present | macrocalc |
| 11 | Microcalcifications | Binary | 1 = present | microcalc |
| 12 | Taller-than-wide orientation | Binary | 1 = taller than wide | taller_than_wide |
