# Supplementary material for: Logistic regression withstands comparison with machine learning for thyroid nodule malignancy risk stratification under strict temporal validation
Source: PLOS Digit Health. 2026 Nov 11;5(11):e0001828. doi: 10.1371/journal.pdig.0001828 (PMC13754167; doi:10.1371/journal.pdig.0001828)
Supplement: Supplementary file 5 [file pdig.0001828.s005.docx]

**S5 Table. Standardised mean differences (SMD) between the training (before 2019) and test (2019 onwards) cohorts.**

*|SMD| > 0.10 indicates a meaningful shift. The largest shifts concern TSH categories, microcalcifications, autoimmune thyroiditis, taller-than-wide orientation, irregular margins and hypoechogenicity; outcome prevalence was stable (SMD +0.008).*

| **Variable** | **SMD (train vs test)** |
| --- | --- |
| Age | -0.062 |
| Family history | +0.036 |
| Male sex | -0.088 |
| TSH suppressed | -0.245 |
| TSH elevated | +0.261 |
| Autoimmune thyroiditis | +0.172 |
| Solid composition | -0.076 |
| Suspicious adenopathies | +0.043 |
| Hypoechogenicity | +0.134 |
| Irregular margins | +0.144 |
| Macrocalcifications | +0.005 |
| Microcalcifications | +0.241 |
| Taller-than-wide | +0.164 |
| Outcome (malignancy) | +0.008 |
