# Supplementary material for: Logistic regression withstands comparison with machine learning for thyroid nodule malignancy risk stratification under strict temporal validation
Source: PLOS Digit Health. 2026 Nov 11;5(11):e0001828. doi: 10.1371/journal.pdig.0001828 (PMC13754167; doi:10.1371/journal.pdig.0001828)
Supplement: Supplementary file 6 [file pdig.0001828.s006.docx]

**S6 Table. Logistic regression coefficients re-estimated in the training cohort (before 2019, n=432), alongside the originally published coefficients.**

*The re-estimated coefficients, together with the intercept, allow full reproduction of individual predictions; they differ from the published coefficients, which were estimated in the derivation cohort (see Methods, 'Handling of the logistic regression model').*

| **Predictor** | **β (training)** | **SE** | **OR (95% CI)** | **β (published) [6,7]** |
| --- | --- | --- | --- | --- |
| (Intercept) | −1.62 | 0.61 | — | −1.55 |
| Age (years) | −0.045 | 0.022 | 0.96 (0.91–1.00) | −0.043 |
| Age² | 0.0006 | 0.0002 | 1.001 (1.000–1.001) | 0.0006 |
| Male sex | 0.46 | 0.27 | 1.59 (0.94–2.69) | 0.49 |
| Family history | 0.58 | 0.49 | 1.78 (0.69–4.62) | 0.61 |
| TSH < 0.369 µU/mL | −0.92 | 0.32 | 0.40 (0.21–0.74) | −0.95 |
| TSH > 4.701 µU/mL | 0.61 | 0.55 | 1.84 (0.63–5.42) | 0.66 |
| Autoimmune thyroiditis | 0.31 | 0.27 | 1.36 (0.80–2.31) | 0.28 |
| Solid composition | 0.74 | 0.30 | 2.10 (1.16–3.78) | 0.71 |
| Suspicious lymph nodes | 1.42 | 0.40 | 4.12 (1.87–9.10) | 1.45 |
| Hypoechoic | 0.78 | 0.24 | 2.18 (1.36–3.49) | 0.81 |
| Irregular/microlobed margins | 1.05 | 0.31 | 2.86 (1.55–5.27) | 1.10 |
| Macrocalcifications | 0.41 | 0.34 | 1.51 (0.78–2.94) | 0.38 |
| Microcalcifications | 1.18 | 0.27 | 3.25 (1.91–5.55) | 1.21 |
| Taller-than-wide | 0.96 | 0.34 | 2.61 (1.34–5.10) | 0.98 |
