# Supplementary material for: Logistic regression withstands comparison with machine learning for thyroid nodule malignancy risk stratification under strict temporal validation
Source: PLOS Digit Health. 2026 Nov 11;5(11):e0001828. doi: 10.1371/journal.pdig.0001828 (PMC13754167; doi:10.1371/journal.pdig.0001828)
Supplement: Supplementary file 7 [file pdig.0001828.s007.docx]

**S1 Checklist. TRIPOD+AI checklist.**

*Based on the official TRIPOD+AI checklist template (Collins GS, Moons KGM, Dhiman P, et al. BMJ 2024;385:e078378. doi:10.1136/bmj-2023-078378; version 11 January 2024). D = items relevant only to the development of a prediction model; E = items relating solely to the evaluation of a prediction model; D;E = items applicable to both. "Reported on page/section" refers to the named sections of the revised manuscript.*

| **Section/Topic** | **Item** | **Development / evaluation** | **Checklist item** | **Reported on page/section** |
| --- | --- | --- | --- | --- |
| **TITLE** | | | | |
| Title | 1 | D;E | Identify the study as developing or evaluating the performance of a multivariable prediction model, the target population, and the outcome to be predicted | Title page (temporal evaluation of LR and ML models; adults with surgically excised thyroid nodules; histologically confirmed malignancy) |
| **ABSTRACT** | | | | |
| Abstract | 2 | D;E | See TRIPOD+AI for Abstracts checklist | Abstract (structured) |
| **INTRODUCTION** | | | | |
| Background | 3a | D;E | Explain the healthcare context (including whether diagnostic or prognostic) and rationale for developing or evaluating the prediction model, including references to existing models | Introduction (diagnostic context; published LR model [6,7]; ML literature [refs]) |
|  | 3b | D;E | Describe the target population and the intended purpose of the prediction model in the context of the care pathway, including its intended users (e.g., healthcare professionals, patients, public) | Introduction; Discussion (clinicians evaluating thyroid nodules pre-operatively) |
|  | 3c | D;E | Describe any known health inequalities between sociodemographic groups | Discussion — limitations (sociodemographic attributes beyond age and sex not recorded) |
| Objectives | 4 | D;E | Specify the study objectives, including whether the study describes the development or validation of a prediction model (or both) | Introduction (final paragraph: head-to-head comparison of re-estimated LR vs four ML algorithms under temporal validation) |
| **METHODS** | | | | |
| Data | 5a | D;E | Describe the sources of data separately for the development and evaluation datasets (e.g., randomised trial, cohort, routine care or registry data), the rationale for using these data, and representativeness of the data | Methods — Study design and participants (single prospectively maintained surgical registry, split temporally) |
|  | 5b | D;E | Specify the dates of the collected participant data, including start and end of participant accrual; and, if applicable, end of follow-up | Methods — Study design and participants; Temporal validation design (development: pre-2019; evaluation: 2019 onwards) |
| Participants | 6a | D;E | Specify key elements of the study setting (e.g., primary care, secondary care, general population) including the number and location of centres | Methods — Study design and participants (one secondary-care centre: Hospital Universitario de Puerto Real, Cádiz, Spain) |
|  | 6b | D;E | Describe the eligibility criteria for study participants | Methods — Study design and participants |
|  | 6c | D;E | Give details of any treatments received, and how they were handled during model development or evaluation, if relevant | Not applicable — all nodules were surgically excised; no treatment effects were modelled |
| Data preparation | 7 | D;E | Describe any data pre-processing and quality checking, including whether this was similar across relevant sociodemographic groups | Methods — Outcome and predictors; Sensitivity analyses (identical pipeline for all participants) |
| Outcome | 8a | D;E | Clearly define the outcome that is being predicted and the time horizon, including how and when assessed, the rationale for choosing this outcome, and whether the method of outcome assessment is consistent across sociodemographic groups | Methods — Outcome and predictors (histologically confirmed malignancy on post-surgical specimen; uniform assessment) |
|  | 8b | D;E | If outcome assessment requires subjective interpretation, describe the qualifications and demographic characteristics of the outcome assessors | Methods — Outcome and predictors (hospital pathologists, routine diagnostic reporting) |
|  | 8c | D;E | Report any actions to blind assessment of the outcome to be predicted | Methods — Outcome and predictors (histopathology reported independently of predictor values as part of routine care) |
| Predictors | 9a | D | Describe the choice of initial predictors (e.g., literature, previous models, all available predictors) and any pre-selection of predictors before model building | Methods — Outcome and predictors (fixed specification of the published LR model [6,7]; no data-driven selection) |
|  | 9b | D;E | Clearly define all predictors, including how and when they were measured (and any actions to blind assessment of predictors for the outcome and other predictors) | Methods — Outcome and predictors; S4 Table (definitions and coding of the twelve predictors / fourteen model terms) |
|  | 9c | D;E | If predictor measurement requires subjective interpretation, describe the qualifications and demographic characteristics of the predictor assessors | Methods — Outcome and predictors (sonographic features assessed by endocrinologists during pre-surgical evaluation) |
| Sample size | 10 | D;E | Explain how the study size was arrived at (separately for development and evaluation), and justify that the study size was sufficient to answer the research question. Include details of any sample size calculation | Methods — Temporal validation design (all eligible registry patients; events-per-variable reported; no formal calculation — discussed as limitation) |
| Missing data | 11 | D;E | Describe how missing data were handled. Provide reasons for omitting any data | Methods — Sensitivity analyses (complete-case primary analysis; MICE sensitivity analysis); Results — Sensitivity analyses; S1 Table |
| Analytical methods | 12a | D | Describe how the data were used (e.g., for development and evaluation of model performance) in the analysis, including whether the data were partitioned, considering any sample size requirements | Methods — Temporal validation design (strict temporal split; no test-set use during development) |
|  | 12b | D | Depending on the type of model, describe how predictors were handled in the analyses (functional form, rescaling, transformation, or any standardisation) | Methods — Outcome and predictors; S4 Table (age + age²; TSH categorised; binary sonographic features) |
|  | 12c | D | Specify the type of model, rationale, all model-building steps, including any hyperparameter tuning, and method for internal validation | Methods — Models and clinical baseline; S2 Table (LR, RF, XGBoost, Bagged CART, MLP; 5-fold CV tuning on training data only) |
|  | 12d | D;E | Describe if and how any heterogeneity in estimates of model parameter values and model performance was handled and quantified across clusters (e.g., hospitals, countries) | Not applicable — single-centre study (acknowledged in Discussion — limitations) |
|  | 12e | D;E | Specify all measures and plots used (and their rationale) to evaluate model performance (e.g., discrimination, calibration, clinical utility) and, if relevant, to compare multiple models | Methods — Performance evaluation (AUC with DeLong tests, PR-AUC, Brier, calibration slope/intercept and plots, decision-curve analysis, operating points) |
|  | 12f | E | Describe any model updating (e.g., recalibration) arising from the model evaluation, either overall or for particular sociodemographic groups or settings | Methods — Models and clinical baseline (out-of-fold isotonic recalibration of ML models on training data; no updating on evaluation data) |
|  | 12g | E | For model evaluation, describe how the model predictions were calculated (e.g., formula, code, object, application programming interface) | Methods — Handling of the logistic regression model; full code at https://github.com/ginejuan/thyroid-ml-vs-lr |
| Class imbalance | 13 | D;E | If class imbalance methods were used, state why and how this was done, and any subsequent methods to recalibrate the model or the model predictions | Methods — Sensitivity analyses; Results — Sensitivity analyses; S1 Table (SMOTE and class-weight sensitivity analyses; not used in primary models) |
| Fairness | 14 | D;E | Describe any approaches that were used to address model fairness and their rationale | Discussion — limitations (protected attributes beyond age and sex unavailable; fairness assessment identified as future work) |
| Model output | 15 | D | Specify the output of the prediction model (e.g., probabilities, classification). Provide details and rationale for any classification and how the thresholds were identified | Methods — Performance evaluation (predicted probabilities; pre-specified 9.55% threshold from the published derivation [6]) |
| Training versus evaluation | 16 | D;E | Identify any differences between the development and evaluation data in healthcare setting, eligibility criteria, outcome, and predictors | Results — Study population; S5 Table (standardised mean differences between cohorts) |
| Ethical approval | 17 | D;E | Name the institutional research board or ethics committee that approved the study and describe the participant-informed consent or the ethics committee waiver of informed consent | Methods — Ethics statement (Biomedical Research Ethics Committee of Cádiz; protocol PAI-TIROIDES-2018) |
| **OPEN SCIENCE** | | | | |
| Funding | 18a | D;E | Give the source of funding and the role of the funders for the present study | Funding (no specific external funding) |
| Conflicts of interest | 18b | D;E | Declare any conflicts of interest and financial disclosures for all authors | Competing interests |
| Protocol | 18c | D;E | Indicate where the study protocol can be accessed or state that a protocol was not prepared | A separate study protocol was not prepared beyond the ethics submission (PAI-TIROIDES-2018) |
| Registration | 18d | D;E | Provide registration information for the study, including register name and registration number, or state that the study was not registered | The study was not registered |
| Data sharing | 18e | D;E | Provide details of the availability of the study data | Data availability (Zenodo, DOI 10.5281/zenodo.22111356, CC BY 4.0) |
| Code sharing | 18f | D;E | Provide details of the availability of the analytical code | Data availability (GitHub, MIT licence) |
| **PATIENT & PUBLIC INVOLVEMENT** | | | | |
| Patient & Public Involvement | 19 | D;E | Provide details of any patient and public involvement during the design, conduct, reporting, interpretation, or dissemination of the study or state no involvement | Methods — Reporting and reproducibility (no patient or public involvement) |
| **RESULTS** | | | | |
| Participants | 20a | D;E | Describe the flow of participants through the study, including the number of participants with and without the outcome and, if applicable, a summary of the follow-up time. A diagram may be helpful | Results — Study population; Figure 1 |
|  | 20b | D;E | Report the characteristics overall and, where applicable, for each data source or setting, including the key dates, key predictors (including demographics), treatments received, sample size, number of outcome events, follow-up time, and amount of missing data. A table may be helpful. Report any differences across key demographic groups | Results — Study population; Table 1; S3 Table (events by subgroup) |
|  | 20c | E | For model evaluation, show a comparison with the development data of the distribution of important predictors (demographics, predictors, and outcome) | Results — Study population; S5 Table (standardised mean differences) |
| Model development | 21 | D;E | Specify the number of participants and outcome events in each analysis (e.g., for model development, hyperparameter tuning, model evaluation) | Results — Study population (training n=432, test n=429, 92 events); S3 Table (subgroup events) |
| Model specification | 22 | D | Provide details of the full prediction model (e.g., formula, code, object, application programming interface) to allow predictions in new individuals and to enable third-party evaluation and implementation, including any restrictions to access or re-use | Methods — Handling of the logistic regression model; S6 Table (re-estimated coefficients); GitHub repository; free web calculator |
| Model performance | 23a | D;E | Report model performance estimates with confidence intervals, including for any key subgroups (e.g., sociodemographic). Consider plots to aid presentation | Results (Tables 2–3; Figures 2–3); Results — Exploratory subgroup analyses |
|  | 23b | D;E | If examined, report results of any heterogeneity in model performance across clusters | Not applicable — single-centre study |
| Model updating | 24 | E | Report the results from any model updating, including the updated model and subsequent performance | Results — Discrimination and calibration (performance of isotonic-recalibrated ML models; Tables 2–3) |
| **DISCUSSION** | | | | |
| Interpretation | 25 | D;E | Give an overall interpretation of the main results, including issues of fairness in the context of the objectives and previous studies | Discussion |
| Limitations | 26 | D;E | Discuss any limitations of the study (such as a non-representative sample, sample size, overfitting, missing data) and their effects on any biases, statistical uncertainty, and generalizability | Discussion — limitations (surgical cohort spectrum, single centre, events-per-variable, missing data, fairness) |
| Usability of the model in the context of current care | 27a | D | Describe how poor quality or unavailable input data (e.g., predictor values) should be assessed and handled when implementing the prediction model | Discussion (implementation: all predictors routinely collected; calculator requires complete predictor entry) |
|  | 27b | D | Specify whether users will be required to interact in the handling of the input data or use of the model, and what level of expertise is required of users | Discussion (intended for clinicians during standard thyroid nodule evaluation) |
|  | 27c | D;E | Discuss any next steps for future research, with a specific view to applicability and generalizability of the model | Discussion; Conclusions (external multi-centre validation, non-surgical spectrum, fairness assessment) |
