# Supplementary material for: Logistic regression withstands comparison with machine learning for thyroid nodule malignancy risk stratification under strict temporal validation
Source: PLOS Digit Health. 2026 Nov 11;5(11):e0001828. doi: 10.1371/journal.pdig.0001828 (PMC13754167; doi:10.1371/journal.pdig.0001828)
Supplement: Supplementary file 8 [file pdig.0001828.s009.docx]

*Peer Review for a Manuscript in PLOS Digital Health*

*Date: 1-August-2026*

**A Validated Logistic Regression Model for Thyroid Nodule Malignancy Risk Stratification Withstands Comparison with Machine Learning Algorithms Under Strict Temporal Validation**

Authors: Prof. Dr. Juan Jesús Fernández Alba *et al*.

Manuscript ID: PDIG-D-26-00938

**These are my comments on the peer review of the submitted manuscript:**

**General comments:**

Overall, this study has clinical value in enhancing the diagnosis of thyroid cancer. The author combined thyroid ultrasound findings and clinical variables constructed in a previous publication. The research analysis method is appropriate, and some additional analyses are recommended to further clarify, such as (1) standardised mean differences (SMDs) between development and temporal validation cohort (2) precision vs. recall analysis to have the full understanding performance of supervised models. The English proficiency of the manuscript text needs to be improved. The paper structure needs improvement, especially in the introduction, discussion, and conclusion sections.

**Specific comments:**

**1. Title**: The title fully describes the study's aims and objectives.

**2. Abstract**: The abstract is acceptable.

**3. Introduction:** The introduction should clearly state the study objectives in the last paragraph of the Introduction section.

“*We conducted a head-to-head comparison of four widely used ML algorithms against our validated LR model under strict temporal validation, with explicit comparison against ATA 2015 risk classification as a clinical baseline, pre-specified subgroup analyses by cytological and sonographic characteristics, and comprehensive sensitivity analyses. The underlying calculator is freely available as a web application at https://obgynreference.shinyapps.io/calccdt/, and all analysis code is publicly released under an open licence*.”

The study objective is not clear in this paragraph. The author wants to compare logistic regression with machine learning models through a validation cohort study based on a previously developed model.

This sentence “*The underlying calculator is freely available as a web application at https://obgynreference.shinyapps.io/calccdt/, and all analysis code is publicly released under an open licence*.”  should be moved to the previous paragraph of the Introduction or the Methods. Currently, this is not appropriately stated and is placed in the last sentence of the Introduction.

**4. Materials and Methods:** Several additional analyses are recommended for the robustness of model comparison.

**-** Comparisons for the 13 predictors and prevalence of outcomes should also be performed in terms of standardised mean differences (SMDs).

**-** I suggest performing an additional analysis of precision versus recall to fully understand the performance of supervised models.

**5. Results:** All the results presented are clear. For consistency and alignment with the study objective of comparing logistic regression and machine learning, the authors are recommended to conduct SHAP analysis built with logistic model, comparing with the best supervised models (either random forest/Xgboost model), to show the robustness of logistic model as authors emphasised.

**6. Discussion:** There is room for English in the discussion, and headings of each paragraph are not required and are redundant; hence, the headings should be omitted.

**7. Conclusion:** The conclusion needs to be rewritten, and the authors should provide a succinct paragraph summarising all the study results. Figures must not be presented again in the Conclusions section.

In conclusion, this study has clinical implications and requires minor revisions for publication.

Thanks,

Best regards,
