# Supplementary material for: Logistic regression withstands comparison with machine learning for thyroid nodule malignancy risk stratification under strict temporal validation
Source: PLOS Digit Health. 2026 Nov 11;5(11):e0001828. doi: 10.1371/journal.pdig.0001828 (PMC13754167; doi:10.1371/journal.pdig.0001828)
Supplement: Supplementary file 9 [file pdig.0001828.s011.docx]

**Response to Editors and Reviewers**

*Manuscript PDIG-D-26-00938 — "Logistic Regression Withstands Comparison with Machine Learning for Thyroid Nodule Malignancy Risk Stratification Under Strict Temporal Validation" (previously: "A Validated Logistic Regression Model for Thyroid Nodule Malignancy Risk Stratification Withstands Comparison with Machine Learning Algorithms Under Strict Temporal Validation")*

Dear Dr. Zuo, Dr. Rappoport, Dr. Celi, and Reviewers,

Thank you for the thorough and constructive evaluation of our manuscript. We have addressed every comment and, in doing so, have substantially strengthened the study. The main changes are:

1. Full transparency on model handling. We now state explicitly that the logistic regression (LR) coefficients were re-estimated in the pre-2019 training cohort using the specification of our previously published model, making the comparison with the machine-learning (ML) algorithms fully symmetric. Accordingly, we no longer describe the analysis as an independent validation of the original model, we disclose the overlap between our cohorts and those of the original derivation and external validation studies, and we have modified the title.

2. Correction of an error. The ATA 2015 AUC of 0.779 reported in the original submission could not be reproduced during this revision; the verified value with the three-level ordinal coding described in Methods is 0.750 (95% CI 0.693–0.806). All ATA-related statements have been corrected, and we now report formal paired DeLong tests: LR, Random Forest, XGBoost and Bagged CART discriminate significantly better than ATA 2015 (p≤0.002), whereas the MLP does not (p=0.45) — which also resolves the internal inconsistency identified by Reviewer 1.

3. New analyses requested by the reviewers, all completed: precision-recall AUC; calibration slope and intercept with bootstrap 95% CIs for all models; operating characteristics of all models at the pre-specified 9.55% threshold (now the primary operating point); standardised mean differences between cohorts; paired DeLong tests versus ATA; learning curves (new S1 Fig); MICE sensitivity analysis extended to all five models; SMOTE and class-weighting sensitivity analyses; SHAP computed on the LR model itself (new Fig 4); an outcome-definition sensitivity analysis excluding incidental microcarcinomas; and event counts for every subgroup (S3 Table).

4. Open data. Beyond the analysis code and synthetic dataset already public, we have deposited the minimal anonymised patient-level dataset (complete-case n=861 and with-missing n=981, with codebook) in the Zenodo repository under a CC BY 4.0 licence, and updated the Data Availability Statement accordingly.

5. Moderated language throughout: conclusions are now explicitly bounded to a single-centre surgical cohort of moderate size with routinely collected structured predictors, and expressions such as "cannot be improved upon", "superior calibration" and "identical predictor hierarchies" have been replaced by more precise wording.

Below we respond point by point. Reviewer comments appear in italics. The revised manuscript includes continuous line numbering; each 'Changes' entry references the corresponding section of the revised manuscript.

**Journal Requirements**

*1. Please ensure that your Ethics Statement is available in its entirety at the beginning of your Methods section, under a subheading 'Ethics Statement'.*

**Response:** Done. The Ethics Statement now opens the Materials and Methods section, naming the Biomedical Research Ethics Committee of Cádiz (Spain; protocol code PAI-TIROIDES-2018, April 2018), the Declaration of Helsinki, and the consent framework (waiver of study-specific consent for registry data owing to the retrospective design; written consent for FNA and surgical procedures).

**Changes:** Methods, new first subsection 'Ethics statement'.

*2. Tables should not be uploaded as individual files. Please include the Tables in your manuscript file as editable, cell-based objects.*

**Response:** Done. All five tables are now embedded in the manuscript as editable cell-based tables, and the individual table files have been removed from the submission. We note with apologies that the previous upload format appears to have prevented at least one reviewer from accessing the tables (Reviewer 5, comment 6).

**Changes:** Tables 1–5 embedded in the manuscript file.

*3. Please upload supplementary files as separate files with the item type 'Supporting Information'.*

**Response:** Done. Supporting Information now comprises S1–S6 Tables, the TRIPOD+AI checklist (S7), and S1 Fig (learning curves), each uploaded with the 'Supporting Information' item type.

**Changes:** Supporting Information files S1–S7 and S1 Fig.

*4. We note that you have indicated that there are restrictions to data sharing for this study… please address the following prompts (a) explain restrictions in detail… (b) if there are no restrictions, please upload the minimal anonymized data set… to a stable, public repository…*

**Response:** We have taken option (b). The minimal anonymised patient-level dataset required to replicate all findings — complete-case (n=861) and with-missing (n=981) versions with a full codebook — is now openly published in Zenodo under a CC BY 4.0 licence: https://doi.org/10.5281/zenodo.22111356 (published 26 August 2026, with the authors' ORCID records and related-work links to the derivation and external-validation articles). The dataset was prepared following the de-identification guidance of Hrynaszkiewicz et al. (BMJ 2010;340:c181): no direct identifiers or dates; surgery year replaced by the analysis cohort label; nodule diameter replaced by the size categories used in the subgroup analyses. The full clinical registry contains additional potentially identifying information and remains restricted. The Data Availability Statement has been updated accordingly.

**Changes:** Data Availability Statement updated; Zenodo deposit.

**Reviewer #1**

We thank the reviewer for a rigorous and constructive review that identified, among other things, a genuine inconsistency in our ATA comparison. All eight points have been addressed.

*1. It is unclear whether the published logistic regression model was applied with its original coefficients and intercept or refitted using the pre-2019 cohort… If the LR model was refitted, the analysis should not be described as an independent validation of the original model.*

**Response:** The reviewer is right to press on this point. The LR coefficients were re-estimated in the pre-2019 training cohort using the predictor specification of the published model; the original coefficients were not applied unchanged. We have added a dedicated Methods subsection ('Handling of the logistic regression model') stating this explicitly, reframed the analysis throughout as a symmetric, temporally validated comparison of algorithms sharing a previously validated specification — not an independent validation of the original locked model — and modified the title accordingly (removing 'A Validated…Model'). We also now disclose that the training period overlaps with the derivation cohort of the original model and the test period with the cohort of its external validation, and list this in Limitations. The re-estimated coefficients are provided in S6 Table and in the public repository.

**Changes:** Title; Methods 'Handling of the logistic regression model'; Abstract; Discussion (Limitations, second point); S6 Table.

*2. The cohort includes only patients who underwent thyroidectomy… Claims regarding immediate clinical deployment or reduction of unnecessary biopsy and surgery should therefore be moderated.*

**Response:** Agreed. We now define the intended target population in Methods (patients considered for surgical referral in comparable settings), state in the Discussion that prevalence, case mix and sonographic spectrum differ from unselected nodule populations, and make explicit that predicted probabilities, decision-curve results and the 9.55% threshold cannot be assumed valid in patients managed with FNAC or surveillance without validation in a representative non-surgical cohort. 'Immediately deployable' has been removed.

**Changes:** Methods 'Study design'; Discussion (implementation paragraph and Limitations, first point); Abstract; Conclusions.

*3. The comparison with ATA 2015 is internally inconsistent. The MLP achieved an AUC of 0.768, which is lower than the ATA AUC of 0.779, yet the manuscript states that all five models outperformed ATA… Pairwise statistical comparisons with ATA should also be reported using the same test population.*

**Response:** We thank the reviewer for catching this genuine error, and we must report a further correction that emerged when we re-ran this analysis: the ATA AUC of 0.779 in the original submission could not be reproduced and was itself incorrect. The verified AUC of the three-level ATA ordinal score on the common test subset (n=426) is 0.750 (95% CI 0.693–0.806). We now report paired DeLong tests for every model against ATA on the same subpopulation: LR, RF and XGBoost p<0.001, Bagged CART p=0.002, and the MLP p=0.45 — that is, four of the five models discriminate significantly better than ATA 2015 and the MLP does not differ significantly from it. All text (Abstract, Results, Discussion, Conclusions) has been corrected, and a sensitivity analysis with the five-level ATA pattern classification yields the same conclusions.

**Changes:** Table 4 (new DeLong column and corrected AUC); Results 'Comparison with ATA 2015'; correction note in the text; Abstract; Conclusions.

*4. The published LR model reportedly uses a 9.55% decision threshold, but sensitivity and specificity are presented at a Youden-derived threshold. A threshold selected from the test cohort is not appropriate as the main operating point in a temporal validation study.*

**Response:** Agreed entirely. The pre-specified 9.55% threshold is now the primary operating point: Table 3 reports sensitivity, specificity, PPV and NPV with bootstrap 95% CIs for all five models at 9.55% (LR: 0.859/0.599/0.369/0.940), alongside ATA 2015 at its binary threshold. Youden-derived values are retained only as clearly labelled post-hoc, descriptive secondary results in the table footnote.

**Changes:** Methods 'Performance evaluation'; new Table 3; Results 'Operating characteristics'.

*5. Calibration reporting should include calibration intercepts and slopes with confidence intervals for all models. Please also clarify whether isotonic calibration was fitted using out-of-fold predictions or fitted predictions from the same training observations.*

**Response:** Both added. Table 2 now reports calibration slope and intercept with 2,000-sample bootstrap 95% CIs for all five models (e.g., LR slope 1.12, 0.91–1.43; XGBoost slope 1.42, 1.10–1.93). Isotonic calibration was implemented with CalibratedClassifierCV (scikit-learn) using 5-fold cross-validation within the training cohort, i.e., the calibration mapping was learned from out-of-fold predictions; no test information was used. This is now stated in Methods.

**Changes:** Table 2 (new columns); Methods 'Models and clinical baseline' and 'Performance evaluation'.

*6. Several subgroup analyses appear underpowered… Please report the number of malignant and benign cases in each subgroup and use more cautious wording.*

**Response:** Done. S3 Table now reports malignant and benign counts for every stratum; the analyses are labelled exploratory in Methods and Results; unstable strata (Bethesda V–VI, n=44, 36 malignant/8 benign; ≤10 mm, n=15, 10 malignant) are explicitly flagged; and all equivalence-type wording has been replaced by 'no evidence of a consistent advantage', with an explicit statement that absence of a significant difference does not establish equivalence.

**Changes:** Methods 'Exploratory subgroup analyses'; Results; S3 Table; Abstract.

*7. The SHAP results are overinterpreted… The comparison between SHAP values and LR coefficients also requires a clearer methodological description.*

**Response:** Agreed. We now describe the comparison operationally in Methods (ranking of mean absolute SHAP values versus ranking of standardised coefficient magnitudes; Spearman correlation; explicitly descriptive), have replaced 'identical predictor hierarchies' with 'broadly similar global orderings', and state that the analysis neither establishes identical hierarchies nor excludes non-linear effects. Additionally — also at the suggestion of Reviewer 2 — we computed SHAP values on the LR model itself, which correlate strongly with XGBoost (ρ=0.88) and RF (ρ=0.69), and redesigned Fig 4 to show the three importance profiles side by side.

**Changes:** Methods 'Predictor importance (SHAP)'; Results; new Fig 4; Discussion.

*8. Please clarify whether each patient contributed only one nodule, how the index nodule was selected, and how incidental thyroid cancers and NIFTP were classified.*

**Response:** Clarified, and the question prompted an informative new sensitivity analysis. Each patient contributed a single index nodule, defined as the most sonographically suspicious nodule at the pre-surgical ultrasound evaluation and recorded as such in the prospective registry; not all index nodules underwent FNA (cytology available in 91.4%). The reference standard was the definitive post-surgical histology of the resected gland, and the primary outcome is specimen-level (any thyroid cancer), consistent with the derivation study, so incidental carcinomas count as events: 16 of the 92 test-cohort events were incidental microcarcinomas. Because the published external validation excluded incidentalomas <1 cm from the outcome, we added a sensitivity analysis excluding incidental microcarcinomas: discrimination improved for every model and LR achieved the highest AUC (0.887), consistent with incidental microcarcinomas being essentially unpredictable from index-nodule characteristics. The registry antedates NIFTP; follicular tumours of uncertain malignant potential (n=4) were classified as non-malignant. All of this is now stated in Methods and Results.

**Changes:** Methods 'Outcome and predictors'; Results 'Sensitivity analyses'.

**Reviewer #2 (Dr. Nguyen Tat Thanh)**

We thank the reviewer for the positive assessment ('requires minor revisions for publication') and the concrete suggestions, all of which we have implemented.

*3. Introduction: The introduction should clearly state the study objectives in the last paragraph… The sentence about the calculator and code should be moved to the Methods.*

**Response:** Done. The Introduction now closes with an explicit aim statement ('The aim of this study was to determine whether…'), and the calculator URL and code availability have been moved to Methods ('Reporting and reproducibility').

**Changes:** Introduction, final paragraph; Methods 'Reporting and reproducibility'.

*4. Materials and Methods: … (1) standardised mean differences (SMDs) between development and temporal validation cohort (2) precision vs. recall analysis…*

**Response:** Both added. S5 Table reports SMDs for all predictors and the outcome; the largest shifts (|SMD|>0.10) are summarised in Methods/Results (TSH categories ±0.25–0.26, microcalcifications +0.24, autoimmune thyroiditis +0.17), with a stable outcome prevalence (SMD 0.008). PR-AUC (average precision) with bootstrap CIs is now reported for all models in Table 2; notably, LR showed the highest PR-AUC (0.672).

**Changes:** S5 Table; Table 2; Methods 'Temporal validation design' and 'Performance evaluation'; Results.

*5. Results: … the authors are recommended to conduct SHAP analysis built with logistic model, comparing with the best supervised models…*

**Response:** An excellent suggestion, implemented in full. SHAP values were computed on the LR model (LinearExplainer) and compared with RF and XGBoost: Spearman ρ=0.69 (RF) and ρ=0.88 (XGBoost), with strong internal consistency against the standardised-coefficient ranking (ρ=0.85). Fig 4 has been redesigned to display the three normalised importance profiles side by side with a rank-agreement panel.

**Changes:** Methods 'Predictor importance (SHAP)'; Results; new Fig 4.

*6. Discussion: headings of each paragraph are not required… should be omitted. / 4. The English proficiency of the manuscript text needs to be improved.*

**Response:** The Discussion headings have been removed and the section now flows as continuous prose. The entire manuscript has undergone a complete language revision.

**Changes:** Discussion; whole manuscript.

*7. Conclusion: The conclusion needs to be rewritten… succinct paragraph… Figures must not be presented again…*

**Response:** The Conclusions have been rewritten as a single succinct paragraph without repeated figures or statistics beyond the essential quantities, and aligned with the moderated framing requested by several reviewers.

**Changes:** Conclusions.

**Reviewer #3 (Ayma Waqar)**

We thank the reviewer for the careful reading and the support for publication after minor revision.

*1. Please clarify the definition of the 13 predictors… the enumeration does not appear to correspond straightforwardly to 13 variables… Figure 4 contains derived features such as age terms and TSH categories.*

**Response:** The reviewer identified a genuine inaccuracy: the previous enumeration ('13 predictors') was a miscount. The model comprises twelve clinical predictors represented by fourteen model terms — exactly replicating Table 1 of the published external validation (including autoimmune thyroiditis, and with age entering linearly and quadratically and TSH as two dummies for suppressed/elevated). New S4 Table lists every predictor with its type, coding and transformation and confirms that the identical design matrix was supplied to all five models. The Methods enumeration has been corrected accordingly.

**Changes:** Methods 'Outcome and predictors'; new S4 Table.

*2. Please clarify how the logistic regression model was handled…*

**Response:** Clarified as described in our response to Reviewer 1, point 1: coefficients re-estimated in the training cohort; explicitly not an independent validation of the locked model; title changed; overlap with previous cohorts disclosed; re-estimated coefficients in S6 Table.

**Changes:** Methods 'Handling of the logistic regression model'; Title; S6 Table.

*3. Consider slightly moderating the wording regarding equivalence… I suggest 'no evaluated ML algorithm demonstrated improved performance over LR'.*

**Response:** Adopted verbatim. This formulation now appears in the Abstract, Results and Conclusions, and 'cannot be improved upon' has been removed throughout.

**Changes:** Abstract; Discussion; Conclusions.

*4. Calibration reporting could be strengthened… calibration intercept and slope…*

**Response:** Added with bootstrap 95% CIs for all models (Table 2); see response to Reviewer 1, point 5.

**Changes:** Table 2.

*5. Please further contextualise the surgical-only cohort…*

**Response:** Done; see response to Reviewer 1, point 2. The Discussion now explicitly addresses generalisability to the broader nodule population and the uncertainty of calibration and clinical utility outside the surgical setting.

**Changes:** Discussion.

*6. The ATA 2015 comparison is useful but could be presented somewhat cautiously… complement rather than replacement…*

**Response:** Maintained and reinforced. The complement-not-replacement framing now appears consistently in Methods, Results, Discussion and Conclusions. Note also the correction of the ATA AUC and the addition of formal DeLong tests (response to Reviewer 1, point 3).

**Changes:** Results; Discussion; Conclusions.

*7. Minor reporting points: consistency of names/coding/sample sizes; complete LR equation; events per subgroup.*

**Response:** All done: a consistency pass has harmonised predictor names and sample sizes across the manuscript, figures and Supporting Information; the complete re-estimated LR equation (intercept and 14 coefficients) is in S6 Table and the repository; and S3 Table now reports malignant/benign counts for every subgroup.

**Changes:** Whole manuscript; S3, S4, S6 Tables.

**Reviewer #4**

We thank the reviewer for a searching methodological review. The seven major questions have shaped much of this revision.

*Q1. The ML models were trained in a cohort of 432 patients… ~7.2 events per predictor… The conclusion should therefore be framed more cautiously… not interpreted as evidence that ML is generally unable to improve clinical prediction.*

**Response:** Fully accepted. The principal conclusion is now explicitly bounded: 'in this single-centre surgical cohort with a moderate sample size and routinely collected structured predictors, none of the evaluated ML algorithms demonstrated improved performance…'. The boundary conditions (larger datasets, high-dimensional inputs such as radiomics, molecular data or imaging) are connected directly to the conclusion in the Discussion. In addition, following Reviewer 6, we performed a learning-curve analysis (new S1 Fig): RF and XGBoost were largely plateaued at the full training size (AUC gain +0.005 and +0.008 from 75% to 100%), suggesting the tree-ensemble result is not merely an artefact of limited data, whereas the MLP was still clearly data-limited — a nuance now discussed explicitly.

**Changes:** Abstract; Discussion (first and fourth paragraphs; Limitations, third point); Conclusions; S1 Fig.

*Q2. The study includes only patients who underwent thyroidectomy… clarify the intended target population… statements suggesting immediate deployment should be moderated… calibration and clinical utility in patients who do not proceed to surgery remain uncertain.*

**Response:** Fully accepted; see response to Reviewer 1, point 2. The intended target population is now defined in Methods, and the Discussion states verbatim that calibration and clinical utility in patients not proceeding to surgery remain uncertain and require validation in a representative non-surgical cohort.

**Changes:** Methods; Discussion; Abstract; Conclusions.

*Q3. Several subgroup analyses contain relatively few patients and events… report events… exploratory rather than confirmatory… avoid implying that ML has been definitively shown to offer no advantage in every subgroup.*

**Response:** Fully accepted; see response to Reviewer 1, point 6. Subgroups are now exploratory, event counts are reported, unstable strata are flagged, and the interpretation is phrased as 'no consistent advantage was observed', with the underpowering acknowledged in the Abstract and Discussion.

**Changes:** Methods; Results; S3 Table; Abstract; Discussion.

*Q4. The ATA 2015 risk category was treated as an ordinal score… explain how the categories were numerically encoded and why… acknowledge that the comparison evaluates the discriminatory information… not the complete ATA clinical decision pathway.*

**Response:** Done. Methods now specify the coding (very low/low=0, intermediate=1, high=2), the rationale (using the full ordinal information rather than only a binary cut), and state that the comparison addresses the discriminatory information of the categories, not the ATA management pathway, which also integrates size and follow-up recommendations. We further clarify that ATA categories were assigned prospectively by the treating endocrinologists during routine pre-surgical evaluation, before surgery and independently of histology. The complement-not-replacement framing is maintained consistently. Please note also the correction of the ATA AUC value and the new formal tests (response to Reviewer 1, point 3).

**Changes:** Methods 'Models and clinical baseline'; Results; Table 4; Discussion; Conclusions.

*Q5. …correlations of 0.71 and 0.77 indicate reasonably strong agreement in global ranking, but they do not establish identical predictor hierarchies… recommend revising the wording… visual comparison… presented as descriptive.*

**Response:** Fully accepted. Wording changed to 'broadly similar global importance orderings'; the comparison is explicitly descriptive; and the new Fig 4 provides precisely the requested visual comparison of normalised importance profiles (now including SHAP computed on LR itself), with a rank-agreement panel.

**Changes:** Methods; Results; new Fig 4; Discussion.

*Q6. 'Superior calibration'… may be stronger than the available evidence supports… provide calibration intercept and slope, ideally with confidence intervals… clarify how isotonic calibration was performed…*

**Response:** Fully accepted. 'Superior calibration' has been replaced by 'better calibration without post-hoc adjustment' (Abstract, Results, Conclusions); slope and intercept with bootstrap CIs are in Table 2; and the isotonic procedure is now described (5-fold CV within training; out-of-fold predictions; no test data).

**Changes:** Table 2; Methods; Abstract; Results; Conclusions.

*Q7. The decision curves appear largely overlapping… the manuscript should distinguish discrimination from clinical utility… decision-curve findings should not be assumed to apply directly to an unselected population.*

**Response:** Both points incorporated: the Results now state that small AUC differences did not translate into net-benefit differences (discrimination vs utility distinction), and the Discussion/Limitations state that decision-curve findings from this surgical cohort cannot be assumed valid in unselected populations.

**Changes:** Results 'Discrimination and calibration'; Discussion.

*Minor Q1. Several references are incomplete… references 6 and 7…*

**Response:** Corrected. The derivation and validation references now include complete bibliographic details and DOIs (Carral San Laureano et al., Endocr Pract 2020;26(10):1077–1084, doi:10.4158/EP-2020-0004; Fernández Alba et al., Diagnostics 2025;15(6):686, doi:10.3390/diagnostics15060686), and the whole reference list has been renumbered consecutively and reformatted.

**Changes:** References (now refs 5 and 6).

*Minor Q2. …wording should consistently indicate that the findings apply to a single-centre surgical cohort, a moderate-sized training sample, and a limited set of structured predictors.*

**Response:** Done throughout; this formulation now appears in the Abstract, the opening of the Discussion and the Conclusions.

**Changes:** Abstract; Discussion; Conclusions.

**Reviewer #5**

We thank the reviewer for a demanding and fair review. We note that the tables were unfortunately not accessible in the submitted files (comment 6), which we believe explains part of the reviewer's reservations; they are now embedded in the manuscript. All eight major comments are addressed below.

*1. …restricting the predictors exclusively to those of LR may inherently limit ML performance… this restricted feature choice could induce an artificial reduction in performance…*

**Response:** This is the one point where we respectfully defend our design, while conceding its implication. The identical predictor set is not an oversight but the central design decision: our research question is whether the learning algorithm improves prediction when the available information is held constant — the apples-to-apples comparison recommended in the comparative literature, since otherwise algorithm effects are confounded with information effects. We agree, however, that this caps the achievable performance of ML pipelines with broader candidate feature pools, and that 'can a richer ML pipeline outperform the published specification?' is a different and legitimate question. We now state the design rationale explicitly in Methods, acknowledge the cap as a limitation (Limitations, fourth point), and identify the broader-pipeline question as future research. Regarding SHAP, we have moderated the interpretation and added SHAP on LR itself precisely so that the comparison is presented as descriptive agreement, not as proof (see responses to Reviewers 1.7 and 4.Q5).

**Changes:** Methods 'Models and clinical baseline'; Discussion (Limitations); Future research statement.

*2. …did you evaluate and compare model performance using PR-AUC…? …provide sensitivity, specificity, PPV, NPV for LR vs. ML as well, rather than solely for ATA 2015…*

**Response:** Both implemented. PR-AUC with bootstrap CIs is reported for all models in Table 2 — LR showed the highest PR-AUC (0.672, 95% CI 0.574–0.758) — and Table 3 now reports sensitivity, specificity, PPV and NPV for all five models at the pre-specified 9.55% threshold, alongside ATA 2015.

**Changes:** Tables 2 and 3; Methods 'Performance evaluation'; Results.

*3. …please provide the completed [TRIPOD+AI] checklist in the supplementary material.*

**Response:** Provided as S7 Checklist.

**Changes:** S7 Checklist.

*4. …did you test ML algorithms using resampling or rebalancing techniques such as SMOTE, ROSE, or Tomek links…?*

**Response:** We have now performed this analysis. RF, XGBoost and the MLP were re-trained after SMOTE oversampling of the training cohort, and with class-weighting (class_weight='balanced' for RF; scale_pos_weight for XGBoost), always evaluating on the intact test cohort. SMOTE increased the RF AUC slightly (0.843→0.858) at the cost of clear calibration deterioration (slope 1.06→0.79; mean predicted risk 0.239 vs observed prevalence 0.214), degraded XGBoost on all metrics (AUC 0.844→0.834; slope 1.42→0.61), left the MLP essentially unchanged in discrimination while worsening its Brier score, and class-weighting produced no improvements. These results are consistent with methodological work showing that imbalance corrections tend to harm risk-prediction calibration (van den Goorbergh et al., JAMIA 2022), now cited.

**Changes:** Methods 'Sensitivity analyses'; Results 'Sensitivity analyses'; S1 Table; new reference 12.

*5. …the sample size is severely constrained for machine learning applications… The absence of ML superiority may reflect a lack of statistical power to train complex models rather than an intrinsic limitation of ML algorithms per se.*

**Response:** We accept the substance of this point and have bounded the conclusion accordingly (see response to Reviewer 4.Q1). The new learning-curve analysis addresses it empirically: the tree ensembles were largely plateaued at the available training size, whereas the MLP was still data-limited. We therefore now distinguish explicitly between the tree-ensemble result (unlikely to reverse with more data of the same kind) and the neural-network result (plausibly data-constrained), and we state that our findings should not be extrapolated to substantially larger datasets.

**Changes:** S1 Fig; Results; Discussion; Limitations.

*6. …the tables do not appear to be accessible in the submitted manuscript files…*

**Response:** We sincerely apologise: the tables were uploaded as separate files following our misreading of the submission format, which evidently prevented access during review. All tables are now embedded in the manuscript as editable objects, per the Journal Requirements.

**Changes:** Tables 1–5 embedded.

*7. …this significant temporal shift illustrates that LR is robust under real-world conditions, [but] it could lead to drastic performance degradation in ML… problematic in a paper whose primary objective is the performance comparison…*

**Response:** An insightful observation. We now quantify the shift with SMDs (S5 Table; largest |SMD| 0.26) and address the point directly in the Discussion: the documented shift did not disproportionately degrade the tree ensembles' discrimination relative to LR (their AUCs were statistically indistinguishable from LR's), although we acknowledge it may have contributed to the MLP's poor performance. We believe this strengthens rather than undermines the comparison: temporal transportability under realistic distribution shift is precisely the property a clinically deployable model must have, and all models faced the identical shift.

**Changes:** S5 Table; Discussion (first paragraph).

*8. In Conclusion: …I would temper this statement. This is a single-center study with a modest sample size and constrained predictors for the ML models.*

**Response:** Done. The Conclusions have been rewritten with the bounded framing ('In a single-centre surgical cohort with a moderate sample size… within these boundary conditions…') and the strong deployment claim has been removed.

**Changes:** Conclusions; Abstract.

*Minor 1. The public GitHub repository contains a number of elements in Spanish…*

**Response:** Done and already published. The repository documentation (README, data-access policy, the docstrings of all analysis scripts) and every figure (ROC, calibration, decision-curve, SHAP) have been translated into English, the citation has been updated to PLOS Digital Health, and the repository now links the open Zenodo dataset (doi:10.5281/zenodo.22111356). The update is live on the main branch of https://github.com/ginejuan/thyroid-ml-vs-lr.

**Changes:** GitHub repository.

*Minor 2. [Christodoulou 2019] I would temper this point. The cited systematic review dates back to 2019…*

**Response:** Accepted. The Introduction now contextualises the review explicitly ('that review predates current reporting standards and many of the weaknesses it documents reflect the practices of the time') and pairs it with more recent methodological literature.

**Changes:** Introduction, third paragraph.

*Minor 3. …citation numbering discrepancy…*

**Response:** The entire reference list has been renumbered consecutively in order of first appearance.

**Changes:** References; all in-text citations.

*Minor 4. There is a growing body of literature demonstrating that for certain tabular clinical datasets… LR match or exceed the performance of complex ML/DL methods. Discussing this literature… would add valuable depth.*

**Response:** Added. The Discussion now engages this literature, citing Grinsztajn et al. (NeurIPS 2022) on tabular data and van den Goorbergh et al. (JAMIA 2022) on imbalance corrections, connected to our empirical findings.

**Changes:** Discussion (fourth paragraph); new references 11 and 12.

**Reviewer #6**

We thank the reviewer for the generous overall assessment and four constructive suggestions, all implemented.

*1. The training sample may be too small for a fully fair comparison with ML methods… I would welcome… learning curves showing AUC against training size, which would help clarify whether the ML models' performance had already plateaued.*

**Response:** Implemented exactly as suggested. Every model was re-fitted on stratified random subsamples of 25%, 50%, 75% and 100% of the training cohort (20 replicates per fraction) and evaluated on the fixed temporal test cohort (new S1 Fig). RF and XGBoost were largely plateaued (mean AUC gain from 75% to 100%: +0.005 and +0.008), whereas the MLP was still clearly ascending (0.663→0.785 from 25% to 100%). The Discussion now acknowledges explicitly that the observed equivalence may partly reflect limited training data — particularly for the neural network — while noting that the plateau makes a data-artefact explanation unlikely for the tree ensembles.

**Changes:** S1 Fig; Methods 'Sensitivity analyses'; Results; Discussion.

*2. The comparison with the ATA 2015 classification would benefit from a formal statistical test… clarify how the ATA 2015 categories were assigned (retrospectively or prospectively) and by whom.*

**Response:** Both done. Paired DeLong tests of every model against the ATA ordinal score on the common subset are now reported (Table 4): LR/RF/XGBoost p<0.001, Bagged CART p=0.002, MLP p=0.45. In verifying this analysis we discovered and corrected an error in the originally reported ATA AUC (0.779 → verified 0.750; see response to Reviewer 1, point 3). ATA categories were assigned prospectively by the treating endocrinologists during routine pre-surgical ultrasound evaluation, before surgery and independently of histology; this is now stated in Methods.

**Changes:** Table 4; Methods 'Models and clinical baseline'; Results.

*3. Verification bias arising from the surgical-only cohort warrants consideration in the clinical-implementation claims…*

**Response:** Accepted; see responses to Reviewers 1.2 and 4.Q2. The implementation paragraph now conditions deployment on comparable populations and explicitly defers the threshold, calibration and decision-curve claims pending validation in the outpatient setting.

**Changes:** Discussion.

*4. …conclusions of 'no ML advantage' in subgroups might better be phrased as 'no evidence of advantage'. In addition, the MICE-based sensitivity analysis appears to have been reported for the LR model only…*

**Response:** Both done. The subgroup phrasing has been adopted verbatim. The MICE analysis (m=20) has been extended to all five models with predictions pooled across imputations: test AUCs 0.831 (LR), 0.839 (RF), 0.845 (XGBoost), 0.810 (Bagged CART) and 0.816 (MLP), reproducing the complete-case pattern; the MLP again showed the largest between-imputation instability (individual-imputation range 0.70–0.84).

**Changes:** Results 'Sensitivity analyses'; S1 Table; Abstract.

**Closing**

We are grateful for the reviewers' time and expertise. Their comments have led to a corrected ATA analysis, eight new or extended analyses, an open patient-level dataset, and a more precise and honest framing of our findings. We hope the revised manuscript now meets the standards of PLOS Digital Health.

Sincerely,

Juan Jesús Fernández Alba, on behalf of all authors

University of Cadiz · INIBICA · Hospital Universitario de Puerto Real
