# Supplementary material for: Logistic regression withstands comparison with machine learning for thyroid nodule malignancy risk stratification under strict temporal validation
Source: PLOS Digit Health. 2026 Nov 11;5(11):e0001828. doi: 10.1371/journal.pdig.0001828 (PMC13754167; doi:10.1371/journal.pdig.0001828)
Supplement: Supplementary file 10 [file pdig.0001828.s012.docx]

**Response to Reviewers — PDIG-D-26-00938R2**

Logistic Regression Withstands Comparison with Machine Learning for Thyroid Nodule Malignancy Risk Stratification Under Strict Temporal Validation

We thank the Academic Editor and the reviewers for their positive assessment and for the remaining editorial suggestions, all of which we have implemented. Reviewer comments are reproduced in italics, followed by our responses. Changes are shown in the Revised Manuscript with Track Changes, which for this round contains only the edits made since the previous (R1) version.

**Reviewer #3**

*"The authors have responded comprehensively to the previous round… I have only two minor, non-blocking editorial points: remove the residual contradictory paragraph in the Data Availability statement, and complete the tracked-changes cleanup (duplicated Abstract phrasing and one lingering “13 predictors”). I recommend acceptance."*

**Response:** Thank you for this recommendation. We believe the three residual items all originate from the R1 tracked-changes file rather than from the clean manuscript: the "contradictory" Data Availability paragraph was the pre-revision statement displayed as struck-through deleted text immediately above the current Zenodo statement; likewise, the duplicated Abstract/Author Summary phrasing and the lingering "13 predictors" were struck-through remnants of the previous version. We have verified that the clean manuscript contains a single, internally consistent Data Availability statement (Zenodo dataset under CC BY 4.0, DOI 10.5281/zenodo.22111356, plus code on GitHub under MIT licence), no duplicated Abstract phrasing, and no occurrence of "13 predictors" — the predictor set is described throughout as twelve clinical predictors represented by fourteen model terms. To remove any ambiguity, the tracked-changes file accompanying this resubmission is a fresh document that marks only the changes made in the current round, so none of the previous round’s deletion markup remains visible.

**Reviewer #5**

*Comment 1 — Line 262: The authors state that "RF showed a similar profile". While this holds true for sensitivity and NPV, Random Forest actually outperforms logistic regression regarding specificity and PPV, with a notable difference of approximately 6% in specificity. I suggest nuancing this statement.*

**Response:** We agree and have revised the sentence to report the operating characteristics explicitly. It now reads: "RF showed comparable sensitivity (0.848, 95% CI 0.771–0.916) and NPV (0.940, 0.909–0.967), with higher specificity (0.656 vs 0.599) and PPV (0.402 vs 0.369) than LR." (Results — Operating characteristics at the pre-specified threshold).

*Comment 2 — S7 Checklist: I recommend using the official TRIPOD+AI checklist template to adhere to standard reporting formats and facilitate readability.*

**Response:** We have rebuilt S7 entirely on the official TRIPOD+AI checklist template (Collins et al., BMJ 2024;385:e078378; version 11 January 2024), preserving the official Section/Topic structure, item numbering (1–27 with sub-items), the Development/Evaluation designations, and the verbatim checklist-item wording, with a final column indicating the manuscript section where each item is reported. While completing the official template we noted that item 19 (patient and public involvement) was not explicitly addressed in the manuscript, so we added a brief statement to Methods — Reporting and reproducibility: "Patients and the public were not involved in the design, conduct, reporting, or dissemination of this study."

We thank the reviewers again for a constructive review process that has substantially improved the manuscript.
