# Supplementary material for: Logistic regression withstands comparison with machine learning for thyroid nodule malignancy risk stratification under strict temporal validation
Source: PLOS Digit Health. 2026 Nov 11;5(11):e0001828. doi: 10.1371/journal.pdig.0001828 (PMC13754167; doi:10.1371/journal.pdig.0001828)
Supplement: Peer review history

# Peer review: Decision Letter 0

**DOI:** 10.1371/journal.pdig.0001828.r001

**Authors:** Nadav Rappoport, Zhichao Zuo

24 Aug 2026

Response to Reviewers Revised Manuscript with Track Changes Manuscript **Journal Requirements:**

1. Please ensure that your Ethics Statement is available in its entirety at the beginning of your Methods section, under a subheading 'Ethics Statement'.

2. Tables should not be uploaded as individual files. Please remove these files and include the Tables in your manuscript file as editable, cell-based objects. For more information about how to format tables, see our guidelines: https://journals.plos.org/digitalhealth/s/tables.

3. Please upload supplementary files as separate files with the item type 'Supporting Information'.

4. We note that you have indicated that there are restrictions to data sharing for this study. For studies involving human research participant data or other sensitive data, we encourage authors to share de-identified or anonymized data. However, when data cannot be publicly shared for ethical reasons, we allow authors to make their data sets available upon request. For information on unacceptable data access restrictions, please see [INSERT LINK]:

https://journals.plos.org/digitalhealth/s/data-availability#loc-unacceptable-data-access-restrictions

Before we proceed with your manuscript, please address the following prompts:

a) If there are ethical or legal restrictions on sharing a de-identified data set, please explain them in detail (e.g., data contain potentially identifying or sensitive patient information, data are owned by a third-party organization, etc.) and who has imposed them (e.g., a Research Ethics Committee or Institutional Review Board, etc.). Please also provide contact information for a data access committee, ethics committee, or other institutional body to which data requests may be sent.

b) If there are no restrictions, please upload the minimal anonymized data set necessary to replicate your study findings to a stable, public repository and provide us with the relevant URLs, DOIs, or accession numbers. Please see http://www.bmj.com/content/340/bmj.c181.long for guidelines on how to de-identify and prepare clinical data for publication. For a list of recommended repositories, please see [INSERT LINK]:

https://journals.plos.org/digitalhealth/s/recommended-repositories

You also have the option of uploading the data as Supporting Information files, but we would recommend depositing data directly to a data repository if possible.

Please update your Data Availability statement in the submission form accordingly.

**Additional Editor Comments (if provided):** **Reviewers' Comments:**

**Comments to the Author**

1. Does this manuscript meet PLOS Digital Health’s publication criteria?

Reviewer #1: Yes

Reviewer #2: Yes

Reviewer #3: Yes

Reviewer #4: Yes

Reviewer #5: Partly

Reviewer #6: Yes

**********

2. Has the statistical analysis been performed appropriately and rigorously?

Reviewer #1: Yes

Reviewer #2: Yes

Reviewer #3: Yes

Reviewer #4: Yes

Reviewer #5: Yes

Reviewer #6: Yes

**********

3. Have the authors made all data underlying the findings in their manuscript fully available (please refer to the Data Availability Statement at the start of the manuscript PDF file)?

The PLOS Data policy

Reviewer #1: Yes

Reviewer #2: Yes

Reviewer #3: Yes

Reviewer #4: No

Reviewer #5: Yes

Reviewer #6: Yes

**********

4. Is the manuscript presented in an intelligible fashion and written in standard English?

Reviewer #1: Yes

Reviewer #2: No

Reviewer #3: Yes

Reviewer #4: Yes

Reviewer #5: Yes

Reviewer #6: Yes

**********

**Review Comments to the Author**

Reviewer #1: This is a clinically relevant study, and the temporal split is more convincing than the internal cross-validation used in many similar comparisons. The use of the same predictors across all models is also appropriate. However, several points need clarification before the main conclusion can be supported.

1. It is unclear whether the published logistic regression model was applied with its original coefficients and intercept or refitted using the pre-2019 cohort. The manuscript states that all five models were trained in the training cohort, while also presenting the LR model as previously validated. Please clarify this point and report whether the present cohort overlaps with the previous derivation or validation cohorts. If the LR model was refitted, the analysis should not be described as an independent validation of the original model.

2. The cohort includes only patients who underwent thyroidectomy. This introduces substantial selection and verification bias, since many patients evaluated for thyroid nodules do not proceed to surgery. Claims regarding immediate clinical deployment or reduction of unnecessary biopsy and surgery should therefore be moderated.

3. The comparison with ATA 2015 is internally inconsistent. The MLP achieved an AUC of 0.768, which is lower than the ATA AUC of 0.779, yet the manuscript states that all five models outperformed ATA. This should be corrected. Pairwise statistical comparisons with ATA should also be reported using the same test population.

4. The published LR model reportedly uses a 9.55% decision threshold, but sensitivity and specificity are presented at a Youden-derived threshold. A threshold selected from the test cohort is not appropriate as the main operating point in a temporal validation study. Results at the prespecified threshold should be reported.

5. Calibration reporting should include calibration intercepts and slopes with confidence intervals for all models. Please also clarify whether isotonic calibration was fitted using out-of-fold predictions or fitted predictions from the same training observations.

6. Several subgroup analyses appear underpowered and have wide confidence intervals. The absence of a statistically significant difference does not establish equivalence. Please report the number of malignant and benign cases in each subgroup and use more cautious wording.

7. The SHAP results are overinterpreted. Correlations of 0.71 and 0.77 indicate general agreement but do not confirm “identical predictor hierarchies” or explain why model performance was similar. The comparison between SHAP values and LR coefficients also requires a clearer methodological description.

8. Please clarify whether each patient contributed only one nodule, how the index nodule was selected, and how incidental thyroid cancers and NIFTP were classified.

Reviewer #2: Peer Review for a Manuscript in PLOS Digital Health

Date: 1-August-2026

A Validated Logistic Regression Model for Thyroid Nodule Malignancy Risk Stratification Withstands Comparison with Machine Learning Algorithms Under Strict Temporal Validation

Authors: Prof. Dr. Juan Jesús Fernández Alba et al.

Manuscript ID: PDIG-D-26-00938

These are my comments on the peer review of the submitted manuscript:

General comments:

Overall, this study has clinical value in enhancing the diagnosis of thyroid cancer. The author combined thyroid ultrasound findings and clinical variables constructed in a previous publication. The research analysis method is appropriate, and some additional analyses are recommended to further clarify, such as (1) standardised mean differences (SMDs) between development and temporal validation cohort (2) precision vs. recall analysis to have the full understanding performance of supervised models. The English proficiency of the manuscript text needs to be improved. The paper structure needs improvement, especially in the introduction, discussion, and conclusion sections.

Specific comments:

1. Title: The title fully describes the study's aims and objectives.

2. Abstract: The abstract is acceptable.

3. Introduction: The introduction should clearly state the study objectives in the last paragraph of the Introduction section.

“We conducted a head-to-head comparison of four widely used ML algorithms against our validated LR model under strict temporal validation, with explicit comparison against ATA 2015 risk classification as a clinical baseline, pre-specified subgroup analyses by cytological and sonographic characteristics, and comprehensive sensitivity analyses. The underlying calculator is freely available as a web application at https://obgynreference.shinyapps.io/calccdt/, and all analysis code is publicly released under an open licence.”

The study objective is not clear in this paragraph. The author wants to compare logistic regression with machine learning models through a validation cohort study based on a previously developed model.

This sentence “The underlying calculator is freely available as a web application at https://obgynreference.shinyapps.io/calccdt/, and all analysis code is publicly released under an open licence.” should be moved to the previous paragraph of the Introduction or the Methods. Currently, this is not appropriately stated and is placed in the last sentence of the Introduction.

4. Materials and Methods: Several additional analyses are recommended for the robustness of model comparison.

- Comparisons for the 13 predictors and prevalence of outcomes should also be performed in terms of standardised mean differences (SMDs).

- I suggest performing an additional analysis of precision versus recall to fully understand the performance of supervised models.

5. Results: All the results presented are clear. For consistency and alignment with the study objective of comparing logistic regression and machine learning, the authors are recommended to conduct SHAP analysis built with logistic model, comparing with the best supervised models (either random forest/Xgboost model), to show the robustness of logistic model as authors emphasised.

6. Discussion: There is room for English in the discussion, and headings of each paragraph are not required and are redundant; hence, the headings should be omitted.

7. Conclusion: The conclusion needs to be rewritten, and the authors should provide a succinct paragraph summarising all the study results. Figures must not be presented again in the Conclusions section.

In conclusion, this study has clinical implications and requires minor revisions for publication.

Thanks,

Best regards,

Reviewer #3: This is a well-structured and clinically relevant study comparing a previously developed logistic regression model with four machine-learning approaches for thyroid nodule malignancy risk prediction under temporal validation. The chronological separation of the training and test cohorts is an important strength, as is the use of the same underlying clinical information across models. Evaluation of discrimination, calibration and decision-curve utility provides a broader comparison than AUC alone.

The principal finding—that the evaluated ML algorithms did not demonstrate a meaningful improvement over logistic regression using these routinely available structured predictors—is interesting and potentially useful for clinical prediction-model development. The manuscript is generally clear, and I have several comments that I believe can be addressed in revision.

1. Please clarify the definition of the 13 predictors.

The Methods describe the model as containing 13 predictors, but the enumeration of age, sex, family history, TSH and eight sonographic characteristics does not appear to correspond straightforwardly to 13 variables. In addition, Figure 4 contains derived features such as age terms and TSH categories.

It would be helpful to provide a concise table listing the 13 original predictors and explaining their coding or transformations. This would make it easier for readers to confirm that equivalent predictor information was supplied to all models.

2. Please clarify how the logistic regression model was handled in the present analysis.

The manuscript describes LR as pre-specified from the previous derivation but also states that the models were trained on the training cohort. Please clarify whether the original LR coefficients and intercept were applied unchanged, recalibrated, or re-estimated using the pre-2019 cohort.

This clarification would help readers distinguish validation of a previously locked model from retraining followed by temporal testing.

3. Consider slightly moderating the wording regarding equivalence between LR and ML.

The results convincingly show that none of the evaluated ML algorithms demonstrated a statistically significant improvement over LR in the temporal test cohort. RF and XGBoost achieved very similar discrimination to LR. However, statements such as LR “cannot be improved upon” may be somewhat stronger than the statistical analysis establishes. I suggest wording this as “no evaluated ML algorithm demonstrated improved performance over LR” or similar. This would preserve the main conclusion while more precisely reflecting the evidence.

4. Calibration reporting could be strengthened.

The inclusion of Brier scores and calibration plots is appreciated. If available, I would encourage the authors to additionally report calibration intercept and calibration slope for the models in the temporal test cohort. This would provide readers with a more complete assessment of calibration.

5. Please further contextualise the surgical-only cohort.

The authors appropriately acknowledge that the cohort consists exclusively of patients who underwent thyroidectomy. Because these patients represent a selected population with a relatively high pre-test probability of malignancy, I suggest briefly expanding the discussion of how this may affect generalisability to the broader population of patients presenting with thyroid nodules.

This does not diminish the value of the comparative model analysis, but it is relevant when discussing eventual point-of-care implementation.

6. The ATA 2015 comparison is useful but could be presented somewhat cautiously.

The finding that the prediction models had higher AUCs than ATA 2015 is clinically interesting. The manuscript reports an ATA AUC of 0.779 compared with approximately 0.84 for the better-performing prediction models. Because ATA 2015 ultrasound classification and multivariable prediction models serve somewhat different purposes, I suggest continuing to frame the LR model as a potential complement to rather than replacement for established ultrasound risk-stratification systems. The Discussion already moves in this direction.

7. Minor reporting points.

Please ensure consistency between the main manuscript, figures and supplementary material regarding predictor names, coding, sample sizes and model specifications. It would also be useful to provide the complete LR equation or a clear reference/location where readers can obtain all coefficients necessary to reproduce individual predictions. For subgroup analyses, reporting the number of malignant events as well as total sample size would help readers interpret the precision of subgroup AUC estimates.

Overall assessment

The manuscript addresses an important issue in clinical prediction modelling and uses a stronger validation strategy than many comparisons of logistic regression and machine learning. The finding that more complex ML algorithms did not provide a clear advantage over a transparent LR model using the same structured clinical predictors is relevant to both researchers and clinicians.

The points above are primarily requests for clarification, improved reporting and moderation of a few conclusions rather than concerns that fundamentally alter the study findings. I would therefore support publication after minor revision.

Reviewer #4: This manuscript addresses a relevant and timely question: whether commonly used machine-learning methods provide meaningful improvement over a conventional logistic regression model for thyroid nodule malignancy prediction. The chronological training–test split, the use of the same predictor set across models, and the consideration of discrimination, calibration, and clinical utility are important strengths. The manuscript is generally well organized, and the main findings are clearly presented. Nevertheless, several methodological and interpretative issues should be addressed before the conclusions can be considered sufficiently robust. Most of these concerns do not invalidate the study, but they require clearer qualification of the findings and, where possible, additional reporting.

Major comments

Q1 The machine-learning models were trained in a cohort of 432 patients, including 94 malignant outcomes. The authors themselves acknowledge that this sample size is limited for the development of data-intensive models and corresponds to approximately 7.2 events per predictor. Although cross-validation and repeated resampling are useful for model tuning and internal stability assessment, they do not fully compensate for limited information in the development sample. The conclusion should therefore be framed more cautiously. The present results support the conclusion that, in this relatively small dataset with a limited number of routinely collected structured predictors, the evaluated machine-learning algorithms did not provide a detectable advantage over logistic regression. They should not be interpreted as evidence that machine learning is generally unable to improve clinical prediction. I suggest revising statements such as “no ML algorithm improved upon a properly specified LR model” to make the data context explicit. The Discussion should also state clearly that the results may not apply to substantially larger datasets or to higher-dimensional inputs such as radiomics, molecular features, or imaging data. The manuscript already briefly recognizes these boundary conditions, but they should be more directly connected to the principal conclusion.

Q2 The study includes only patients who underwent thyroidectomy and therefore had histopathological confirmation. This is an important source of verification and selection bias because most patients with thyroid nodules in routine practice do not undergo surgery. The prevalence, case mix, and spectrum of sonographic findings in a surgical cohort are likely to differ from those in an unselected thyroid nodule population. This limitation has particular implications for calibration and clinical applicability. Even if discrimination remains stable, the predicted probabilities may not be directly transferable to patients managed with FNAC or ultrasound surveillance. The authors should therefore clarify the intended target population and prediction setting. Statements suggesting immediate deployment in routine thyroid nodule assessment should be moderated unless the model has also been validated in a representative non-surgical cohort. The Discussion should explicitly state that calibration and clinical utility in patients who do not proceed to surgery remain uncertain and require further external validation.

Q3 Several subgroup analyses contain relatively few patients and events. For example, the Bethesda V–VI subgroup includes only 44 patients, and the reported AUC estimates have wide confidence intervals. In this situation, the absence of an observed machine-learning advantage may reflect insufficient precision rather than genuine similarity between models. Please report the number of malignant and non-malignant outcomes within each subgroup, not only the total sample size. The manuscript should clearly identify subgroups in which performance estimates are unstable because of limited events. These analyses would be better presented as exploratory rather than confirmatory. The abstract and Discussion should also avoid implying that machine learning has been definitively shown to offer no advantage in every clinical subgroup. A more appropriate interpretation would be that no consistent advantage was observed, while acknowledging that several subgroup comparisons were underpowered.

Q4 The ATA 2015 risk category was treated as an ordinal score and analysed as a continuous variable for AUC estimation. This is statistically possible, but it does not fully correspond to how ATA recommendations are applied in clinical practice, where risk categories are interpreted together with nodule size and management thresholds. The authors should explain more clearly how the ATA categories were numerically encoded and why this approach was selected. They should also acknowledge that the comparison evaluates the discriminatory information contained in the ATA risk categories rather than the complete ATA clinical decision pathway. The manuscript appropriately notes that ATA and TI-RADS systems have broader purposes, including standardized ultrasound reporting and management guidance. This distinction should be maintained consistently throughout the Results, Discussion, and Conclusion so that the prediction model is not presented as a direct replacement for a clinical guideline.

Q5 The authors report Spearman correlations of 0.71 and 0.77 between SHAP importance rankings and standardized logistic regression coefficients and interpret these results as confirmation that the models identified the same predictors with the same hierarchy. These correlations indicate reasonably strong agreement in global ranking, but they do not establish identical predictor hierarchies or equivalence in effect magnitude. Rank correlation also does not demonstrate that the machine-learning models failed to capture non-linearities or interactions. I recommend revising the wording to state that the models showed broadly similar global importance rankings. The authors could also provide a visual comparison of normalized absolute logistic regression coefficients and mean absolute SHAP values. However, any such comparison should be presented as descriptive because these quantities are not directly equivalent.

Q6 The manuscript states that logistic regression demonstrated “superior calibration,” based primarily on the Brier score and the relative stability observed across resampling procedures. The finding is potentially important, but “superior” may be stronger than the available evidence supports, particularly when some machine-learning models were recalibrated and the absolute differences appear modest. Please provide, or more prominently report, standard calibration measures such as calibration intercept and calibration slope, ideally with confidence intervals. It would also be helpful to clarify how isotonic calibration was performed and whether the calibration procedure relied exclusively on training data or out-of-fold predictions. Unless the differences are clearly clinically meaningful and statistically supported, wording such as “better calibrated” or “more stable calibration” would be more appropriate than “superior calibration.” The corresponding language in the Abstract and Conclusion should be revised consistently.

Q7 The decision curves appear largely overlapping across the clinically relevant threshold range, and the authors conclude that the machine-learning models did not provide additional net clinical benefit. This interpretation is reasonable, but the clinical meaning should be explained more explicitly. In particular, the manuscript should distinguish discrimination from clinical utility. Small differences in AUC do not necessarily translate into meaningful differences in net benefit. This point would strengthen the practical interpretation of the results. At the same time, because the analysis was performed in a selected surgical cohort, the decision-curve findings should not be assumed to apply directly to an unselected thyroid nodule population.

Minor comments

Q1 Several references are incomplete or inconsistently formatted. In particular, references 6 and 7 do not provide full bibliographic information in the current version. Please revise the reference list according to the journal’s formatting requirements and verify that all cited studies include complete publication details.

Q2 The main message is clinically useful, but the wording should consistently indicate that the findings apply to a single-centre surgical cohort, a moderate-sized training sample, and a limited set of structured predictors. Statements implying universal equivalence between logistic regression and machine learning should be avoided.

Reviewer #5: The manuscript is very well written, clear, and concise. The clinical rationale, methodology, and main conclusions are easy to understand and well articulated.

Major Comments

1. In the Methods section, the authors state: "Patients were excluded if fewer than nine of the 13 model predictors were available.", "they were not included as model predictors to preserve comparability with the published derivation", and "High correlation would indicate that ML algorithms are capturing the same clinical signal as LR — a convergent-validity argument that addresses the common objection that ML models may exploit non-linear interactions invisible to LR." In my view, this represents the main methodological weakness. While I understand that the objective is a strict head-to-head comparison with LR, restricting the predictors exclusively to those of LR may inherently limit ML performance. Standard practice typically involves starting with a broad candidate feature pool followed by feature selection to retain the most influential variables in the final model. Similarly, when performing SHAP analysis, forcing this constrained predictor subset may artificially force the ML models to assign importance to the exact same predictors as LR. This restricted feature choice could induce an artificial reduction in performance, which would undermine your overall conclusions and the primary message of your article.

2. In the "Methods - Performance evaluation" paragraph, regarding "discrimination — AUC, ", given the imbalanced dataset, did you evaluate and compare model performance using PR-AUC rather than ROC-AUC, which is less susceptible to performance overestimation in this type of dataset? Furthermore, in the same paragraph, when stating "sensitivity,specificity, PPV, and NPV were computed at the standard binary threshold", it would be relevant to provide these metrics for LR vs. ML as well, rather than solely for ATA 2015, in order to go beyond global AUC metrics.

3. In Methods, it is noted: "The study is reported in full accordance with the TRIPOD+AI 2024 statement". This is commendable; however, to objectify this compliance, please provide the completed checklist in the supplementary material.

4. In Results: "The training cohort comprised 432 patients (94 malignant, 21.8%) and the test cohort 429 patients (92 malignant, 21.4%). " Given this imbalanced dataset, did you test ML algorithms using resampling or rebalancing techniques such as SMOTE, ROSE, or Tomek links, which could potentially improve performance in this context?

5. Regarding sample size and statistical power: In Results, you report that "The training cohort comprised 432 patients (94 malignant, 21.8%) and the test cohort 429 patients (92 malignant, 21.4%)." With only 94 events (malignant cases) in the training set for 13 predictors (~7 events per variable, EPV), the sample size is severely constrained for machine learning applications. According to current methodological standards for clinical prediction models (e.g., Riley et al., BMJ), 94 events are barely sufficient to fit a standard multivariable logistic regression without overfitting risk. Complex non-linear architectures such as XGBoost, Random Forest, or MLP typically require hundreds or thousands of events to effectively learn complex interactions without overfitting. The absence of ML superiority in this study may therefore reflect a lack of statistical power to train complex models rather than an intrinsic limitation of ML algorithms per se.

6. In Results: Unless I am mistaken, the tables do not appear to be accessible in the submitted manuscript files, which precludes a substantial part of the peer review process.

7. In Results: "Temporal shifts were observed in TSH distribution (TSH <0.369 µU/mL: 18.8% training vs. 29.1% test, p<0.001), nodule size (>40 mm: 17.6% vs. 28.4%, p=0.002), microcalcifications (19.0% vs. 10.5%, p<0.001)," While this significant temporal shift illustrates that LR is robust under real-world conditions, it could lead to drastic performance degradation in ML. If the training set diverges significantly from the test set, ML performance may be disproportionately impaired, which is problematic in a paper whose primary objective is the performance comparison between LR and ML.

8. In Conclusion: " For clinicians and healthcare institutions, the practical message is clear: a validated, transparent logistic regression model with routine clinical and sonographic predictors is sufficient for thyroid nodule malignancy risk stratification, and may be preferable for clinical deployment under emerging regulations on medical artificial intelligence." I would temper this statement. This is a single-center study with a modest sample size and constrained predictors for the ML models.

Minor Comments

The public GitHub repository (https://github.com/ginejuan/thyroid-ml-vs-lr) contains a number of elements (figure legends, code documentation, etc.) in Spanish, which may hinder readability for non-Spanish readers.

2. In the Introduction: "he systematic review by Christodoulou and colleagues [11] found no overall advantage of ML over LR across71 published clinical prediction studies, but highlighted that the comparative evidence base is dominated by methodologically weak studies." I would temper this point. The cited systematic review dates back to 2019, when reporting standards and guidelines were less explicit, which may explain those methodological weaknesses. If the same review were conducted today, these methodologically weak studies would likely not pass peer review. Furthermore, ML may be intrinsically comparable or inferior to LR depending on specific prediction goals and tabular dataset structures.

3. In the "Methods - Performance evaluation" paragraph, there appears to be a citation numbering discrepancy, jumping from reference 11 in the Introduction to references 18-19 here.

4. There is a growing body of literature demonstrating that for certain tabular clinical datasets and prediction tasks, classical methodologies such as LR match or exceed the performance of complex ML/DL methods. Discussing this literature in greater detail would add valuable depth to your Discussion section.

Reviewer #6: Overall assessment

This is a well-conducted and clearly written study that compares a validated logistic regression (LR) model with four machine-learning (ML) algorithms for risk stratification of thyroid nodule malignancy under strict temporal validation. The methodology is rigorous, reporting follows the TRIPOD+AI guidance, and the analysis code together with a synthetic dataset are openly available. I find the study clinically relevant and the main conclusion convincing: in this setting, no ML algorithm meaningfully improved upon a well-specified LR model. I have a few comments that I would ask the authors to consider.

Comments

1. The training sample may be too small for a fully fair comparison with ML methods. The ML algorithms were trained on 432 patients (94 events; EPV ≈ 7.2), which is below the recommended minimum for prediction modelling and, as the authors themselves note, ML methods generally require substantially larger datasets. The observed equivalence between ML and LR may therefore partly reflect limited training data rather than a true equivalence of the methods. I would welcome a more explicit acknowledgement of this point in the Discussion and, if feasible, learning curves showing AUC against training size, which would help clarify whether the ML models' performance had already plateaued.

2. The comparison with the ATA 2015 classification would benefit from a formal statistical test. The confidence intervals for ATA 2015 (0.691–0.812) and for the four models (0.789–0.884) overlap, and no significance test is reported for this comparison. Reporting a DeLong test (or equivalent) of ATA 2015 against each model would strengthen the claim that the models “substantially outperformed” ATA 2015. In addition, because systematic TI-RADS scoring was not available in the early years of the registry, it would be helpful to clarify how the ATA 2015 categories were assigned (retrospectively or prospectively) and by whom.

3. Verification bias arising from the surgical-only cohort warrants consideration in the clinical-implementation claims. As the authors acknowledge, only patients who underwent thyroidectomy were included, so the calibration, the decision-curve analysis, and the 9.55% probability threshold were all derived from a surgically selected population. A brief discussion of how these findings translate to the broader outpatient setting, where most nodules are managed conservatively, would strengthen the clinical-implementation section.

4. Subgroup null results and sensitivity analyses. The subgroup analyses are valuable, but some strata are small (e.g., Bethesda III–IV, n = 79), and conclusions of “no ML advantage” in subgroups might better be phrased as “no evidence of advantage”. In addition, the MICE-based sensitivity analysis appears to have been reported for the LR model only; it would be useful to state whether all models were evaluated under imputation.

These are, in my view, minor-to-moderate points. Overall, the study makes a valuable contribution.

**********

what does this mean?). If published, this will include your full peer review and any attached files.

**Do you want your identity to be public for this peer review?**  If you choose “no”, your identity will remain anonymous but your review may still be made public.

For information about this choice, including consent withdrawal, please see our Privacy Policy

Reviewer #1: No

Reviewer #2: **Yes:**  Nguyen Tat Thanh, MD PhD

Reviewer #3: **Yes:**  Ayma Waqar

Reviewer #4: No

Reviewer #5: No

Reviewer #6: No

**********

**Figure resubmission:**

**Reproducibility:**

To enhance the reproducibility of your results, we recommend that authors of applicable studies deposit laboratory protocols in protocols.io, where a protocol can be assigned its own identifier (DOI) such that it can be cited independently in the future. Additionally, PLOS ONE offers an option to publish peer-reviewed clinical study protocols. Read more information on sharing protocols at https://plos.org/protocols?utm_medium=editorial-email&utm_source=authorletters&utm_campaign=protocols

Attachment Submitted filename: Peer-Review-PLOS Digital Health-1Aug2026.docx

# Author comment: Author response to Decision Letter 1

**DOI:** 10.1371/journal.pdig.0001828.r002

27 Aug 2026

Attachment Submitted filename: Response_to_Reviewers.docx

# Peer review: Decision Letter 1

**DOI:** 10.1371/journal.pdig.0001828.r003

**Authors:** Tim M.H. Li, Zhichao Zuo

6 Oct 2026

Response to ReviewersRevised Manuscript with Track ChangesManuscript**Journal Requirements:** **Reviewers' Comments:**

**Comments to the Author**

Reviewer #3: All comments have been addressed

Reviewer #4: (No Response)

Reviewer #5: (No Response)

**********

publication criteria?

Reviewer #3: Yes

Reviewer #4: (No Response)

Reviewer #5: Yes

**********

3. Has the statistical analysis been performed appropriately and rigorously?

Reviewer #3: Yes

Reviewer #4: (No Response)

Reviewer #5: Yes

**********

4. Have the authors made all data underlying the findings in their manuscript fully available (please refer to the Data Availability Statement at the start of the manuscript PDF file)?

The PLOS Data policy

Reviewer #3: Yes

Reviewer #4: (No Response)

Reviewer #5: Yes

**********

5. Is the manuscript presented in an intelligible fashion and written in standard English?

Reviewer #3: Yes

Reviewer #4: (No Response)

Reviewer #5: Yes

**********

**Reviewer #3:**  The authors have responded comprehensively to the previous round. All of my earlier comments have been addressed, including the predictor enumeration, the clarified handling of the logistic regression model, the strengthened calibration reporting, the expanded discussion of generalizability beyond the surgical cohort, and the moderated equivalence language. The correction of the ATA AUC and the addition of formal DeLong tests further strengthen the paper. I have only two minor, non-blocking editorial points: remove the residual contradictory paragraph in the Data Availability statement, and complete the tracked-changes cleanup (duplicated Abstract phrasing and one lingering "13 predictors"). I recommend acceptance.

**Reviewer #4:**  (No Response)

**Reviewer #5** : I thank the authors for their thorough responses to the previous comments. I now have only two minor formatting points remaining.

Comment 1:

Line 262: The authors state that "RF showed a similar profile". While this holds true for sensitivity and NPV, Random Forest actually outperforms logistic regression regarding specificity and PPV, with a notable difference of approximately 6% in specificity. I suggest nuancing this statement to accurately reflect these differences rather than describing the performance profiles as entirely similar.

Comment 2:

S7 Checklist: Although the checklist appears comprehensive, its current manual layout makes it somewhat difficult to navigate and assess. I recommend using the official TRIPOD+AI checklist template (available at: https://www.bmj.com/content/bmj/suppl/2024/04/17/bmj-2023-078378.DC5/colg078378.wt2.pdf) to adhere to standard reporting formats and facilitate readability.

**********

what does this mean?). If published, this will include your full peer review and any attached files.

**Do you want your identity to be public for this peer review?**  If you choose “no”, your identity will remain anonymous but your review may still be made public.

For information about this choice, including consent withdrawal, please see our Privacy Policy

Reviewer #3: No

Reviewer #4: None

Reviewer #5: No

**********

**Figure resubmission:**

**Reproducibility:**

To enhance the reproducibility of your results, we recommend that authors of applicable studies deposit laboratory protocols in protocols.io, where a protocol can be assigned its own identifier (DOI) such that it can be cited independently in the future. Additionally, PLOS ONE offers an option to publish peer-reviewed clinical study protocols. Read more information on sharing protocols at https://plos.org/protocols?utm_medium=editorial-email&utm_source=authorletters&utm_campaign=protocols

# Author comment: Author response to Decision Letter 2

**DOI:** 10.1371/journal.pdig.0001828.r004

7 Oct 2026

Attachment Submitted filename: Response_to_Reviewers_auresp_2.docx

# Editor report: Decision Letter 2

**DOI:** 10.1371/journal.pdig.0001828.r005

**Authors:** Tim M.H. Li, Zhichao Zuo

28 Oct 2026

Logistic Regression Withstands Comparison with Machine Learning for Thyroid Nodule Malignancy Risk Stratification Under Strict Temporal Validation

PDIG-D-26-00938R2

Dear Juan Jesús Fernández Alba,

We are pleased to inform you that your manuscript 'Logistic Regression Withstands Comparison with Machine Learning for Thyroid Nodule Malignancy Risk Stratification Under Strict Temporal Validation' has been provisionally accepted for publication in PLOS Digital Health.

Before your manuscript can be formally accepted you will need to complete some formatting changes, which you will receive in a follow-up email from a member of our team.

Please note that your manuscript will not be scheduled for publication until you have made the required changes, so a swift response is appreciated.

IMPORTANT: The editorial review process is now complete. PLOS will only permit corrections to spelling, formatting or significant scientific errors from this point onwards. Requests for major changes, or any which affect the scientific understanding of your work, will cause delays to the publication date of your manuscript.

If your institution or institutions have a press office, please notify them about your upcoming paper to help maximize its impact. If they'll be preparing press materials, please inform our press team as soon as possible -- no later than 48 hours after receiving the formal acceptance. Your manuscript will remain under strict press embargo until 2 pm Eastern Time on the date of publication. For more information, please contact digitalhealth@plos.org.

Thank you again for supporting Open Access publishing; we are looking forward to publishing your work in PLOS Digital Health.

Best regards,

Zhichao Zuo

Academic Editor

PLOS Digital Health

***********************************************************

**Additional Editor Comments (if provided):**

**Reviewer Comments (if any, and for reference):**
